# *FUSE* binding protein 1 (FUBP1) expression is upregulated by T-cell acute lymphocytic leukemia protein 1 (TAL1) and required for efficient erythroid differentiation

**DOI:** 10.1371/journal.pone.0210515

**Published:** 2019-01-17

**Authors:** Marlene Steiner, Lucas Schneider, Jasmin Yillah, Katharina Gerlach, Olga N. Kuvardina, Annekarin Meyer, Alisa Maring, Halvard Bonig, Erhard Seifried, Martin Zörnig, Jörn Lausen

**Affiliations:** 1 Georg-Speyer-Haus, Institute for Tumor Biology and Experimental Therapy, Frankfurt/Main, Germany; 2 Institute for Transfusion Medicine and Immunohematology, Goethe-University and German Red Cross Blood Service, Frankfurt am Main, Germany; Wayne State University, UNITED STATES

## Abstract

During erythropoiesis, haematopoietic stem cells (HSCs) differentiate in successive steps of commitment and specification to mature erythrocytes. This differentiation process is controlled by transcription factors that establish stage- and cell type-specific gene expression. In this study, we demonstrate that *FUSE* binding protein 1 (FUBP1), a transcriptional regulator important for HSC self-renewal and survival, is regulated by T-cell acute lymphocytic leukaemia 1 (TAL1) in erythroid progenitor cells. TAL1 directly activates the *FUBP1* promoter, leading to increased *FUBP1* expression during erythroid differentiation. The binding of TAL1 to the *FUBP1* promoter is highly dependent on an intact GATA sequence in a combined E-box/GATA motif. We found that FUBP1 expression is required for efficient erythropoiesis, as FUBP1-deficient progenitor cells were limited in their potential of erythroid differentiation. Thus, the finding of an interconnection between GATA1/TAL1 and FUBP1 reveals a molecular mechanism that is part of the switch from progenitor- to erythrocyte-specific gene expression. In summary, we identified a TAL1/FUBP1 transcriptional relationship, whose physiological function in haematopoiesis is connected to proper erythropoiesis.

## Introduction

Every second about two million mature red blood cells (erythrocytes) are produced in the bone marrow of a human adult and released into the blood stream to allow the continuous supply with oxygen of all tissues [1]. Along their route to becoming an erythrocyte, haematopoietic stem cells (HSCs) undergo successive steps of specification, commitment and differentiation to produce mature red blood cells. In the classic erythropoiesis model, this differentiation path is hierarchically organized, with multipotent HSCs at the top of the hierarchy. The subsequent steps of differentiation chronologically comprise of multipotent progenitors (MPPs), committed common myeloid progenitors (CMPs) and bipotent megakaryocyte-erythroid progenitors (MEPs) [2, 3]. While a gradual loss of differentiation potential during this pathway is observed *in vitro*, recent *in vivo* and *in silico* analysis revealed that both MPPs and CMPs can already be destined to become an erythrocyte, challenging the classic hierarchical model [4, 5]. Differentiation stages downstream of MEPs are characterized by distinct cell morphology and surface markers [6].

Every fate decision in the pathway of erythropoiesis is ultimately executed by transcription factors, which control the up- and downregulation of lineage- and stage-specific gene expression. Transcription factors that are known to be essential for normal erythropoiesis include TAL1, LMO2, GATA2 and v-MYB which control differentiation of multipotent progenitors, as well as lineage specific factors like GATA1, FOG, and KLF-family members. The different functions of these factors are summarized in *Perry et al*, 2002 [7]. TAL1, GATA1, LMO2 and c-MYC have been identified as minimal factors that are required for the direct lineage programming of fibroblasts into erythroid progenitors [8]. Genome-wide DNA binding site profiling has shown that TAL1, GATA1 and LMO2 often function cooperatively with other transcription factors, such as the haematopoietic master regulator KLF1. Furthermore they are present in multi-protein complexes, including adapter protein LDB1 [9, 10]. The interplay of these protein complexes, the dynamics of complex compositions and their exact function on target genes–including a complete list of targets–remain to be elucidated.

We recently identified Fubp1 (FUSE binding protein 1) as an essential regulator of murine long-term HSC (LT-HSC) self-renewal and survival. Besides its important role for HSCs, we demonstrated that Fubp1 also impacts erythropoiesis, as mice with a functional inactivation of *Fubp1* have less erythroid progenitors in the fetal liver [11] and murine embryonic stem cells display a reduced potential to differentiate into erythrocytes upon *Fubp1* knockout [12].

FUBP1 is a transcriptional regulator that was originally identified as a factor binding the *FUSE* motif in the promoter of the oncogene *c-MYC*. *FUSE* bound FUBP1 interacts with basal transcription factor IIH, inducing helicase activity and promoter escape of the polymerase complex. This results in a peak expression of *c-MYC* mRNA [13]. Besides *c-MYC*, only the cell cycle inhibitor *p21*, apoptosis inducer *BIK* and *ubiquitin specific peptidase 29* possess a known *FUSE* sequence motif in the promoter region and are regulated by FUBP1 [14, 15], but other targets of FUBP1 have been identified, among them *Noxa*, *Cyclin D2*, *p53* and *p27* [14, 16, 17]. FUBP1 regulates the expression of multiple targets involved in migration, apoptosis and proliferation. However, factors that control *FUBP1* expression in general and during haematopoietic differentiation in particular are mostly unknown.

In this study, we addressed the question of upstream regulation of *FUBP1* and identified TAL1 and GATA1 as regulators of *FUBP1* expression in erythroid progenitors. We could show that in primary hCD34^+^ cells, increased TAL1 and GATA1 occupancy of the FUBP1 promoter is observed during erythroid differentiation. This correlates with increased FUBP1 expression in early erythropoiesis. Loss of FUBP1 activity decreased erythropoiesis of hCD34^+^ cells. Concluding from our data, FUBP1 plays an important role in efficient differentiation of human erythrocytes.

## Results

### *FUBP1* and *TAL1* are co-expressed in cell lines and during erythroid differentiation of human primary CD34^+^ haematopoietic progenitor cells

**Fig 1:**
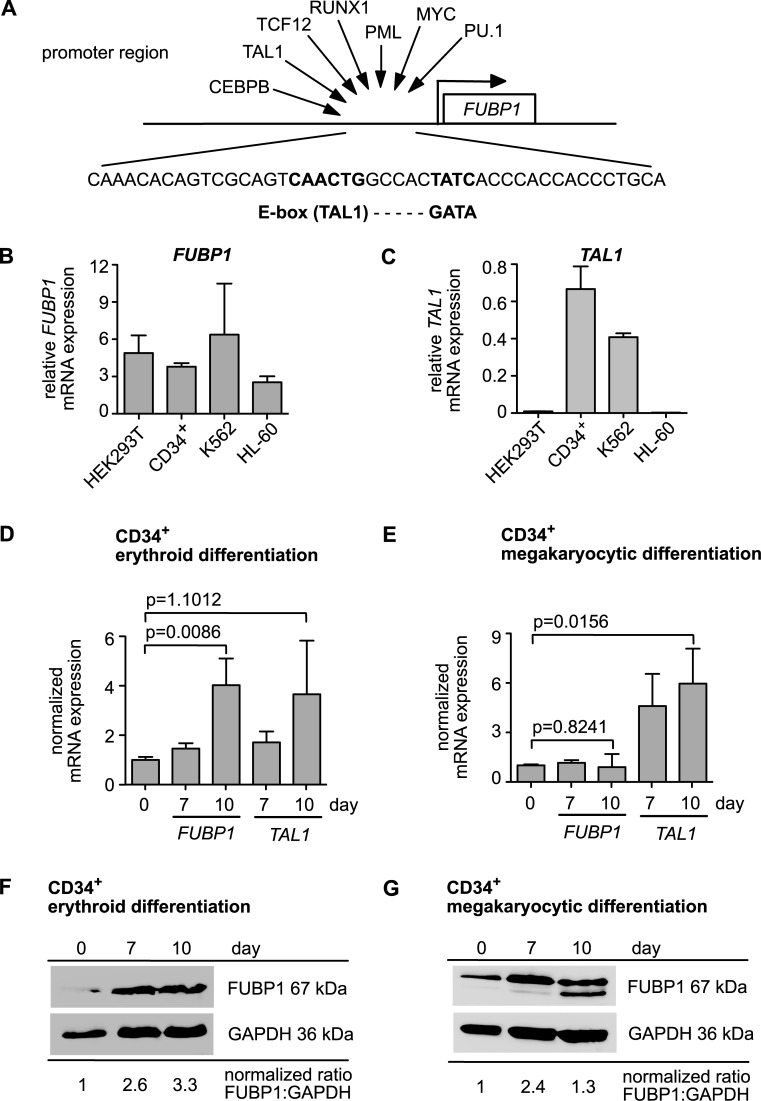
*FUBP1* and *TAL1* co-expression increases during erythroid differentiation. **(A)** The *in silico* analysis of the *FUBP1* promoter revealed several DNA binding motifs for transcription factors. Schematic representation of the *FUBP1* locus is shown. Transcription factors are depicted, which could bind to the promoter region. Close to the transcriptional start site, an *E-box* and a juxtaposed *GATA* element are located, which represent potential binding sites for TAL1 and GATA1. **(B)** *FUBP1* mRNA expression in human embryonic kidney cells (HEK293T), human primary CD34^+^ cells, K562 cells and HL-60 cells. **(C)** *TAL1* mRNA expression in HEK293T, human primary CD34^+^ cells, K562 cells and HL-60 cells. **(D)** *FUBP1* and *TAL1* mRNA expression upon erythroid differentiation of human primary CD34^+^ cells. Relative mRNA expression levels seven and ten days after induction of differentiation, are represented as fold change over the expression at day zero. **(E)** *FUBP1* and *TAL1* mRNA expression during megakaryocytic differentiation of hCD34^+^ cells. Relative expression levels seven and ten days after induction of differentiation are shown as fold change over the expression at day 0. **(F)** FUBP1 protein expression upon erythroid differentiation of primary hCD34^+^ cells. GAPDH was detected as loading control and the ratio of quantified FUBP1 and GAPDH signals is annotated. The dashed line marks where the blot was cut to incubate the upper part with anti-FUBP1 antibody and the lower part with anti-GAPDH antibody. **(G)** FUBP1 protein expression upon megakaryocytic differentiation of primary hCD34^+^ cells. GAPDH was detected as loading control and the ratio of quantified FUBP1 and GAPDH signals is annotated. See also **S5 Fig.** mRNA levels were evaluated by quantitative real-time PCR. Values were normalized according to *GAPDH* expression levels. The error bars reflect mean values with SD of three independent experiments.

In our previous studies, we discovered an important role for FUBP1 in haematopoiesis, namely for the self-renewal of LT-HSCs and during erythroid differentiation of murine embryonic stem cells [11, 12]. Since nothing was known about the upstream regulation of FUBP1 and the factors that control FUBP1 expression levels during haematopoietic differentiation, we were interested to identify proteins that are involved in the transcriptional control of *FUBP1* in blood cells. We analysed *in silico* the human *FUBP1* promoter region for DNA binding motifs recognized by transcription factors and found, amongst others, an E-box followed by a GATA-box (**Fig 1A**). GATA-1- or GATA-2-containing multiprotein complexes at these composite elements control the transcription of genes critical for erythroid cell maturation. Other constituents of the complex include the E-box-binding basic helix-loop-helix transcription factor TAL1, its heterodimerisation partner E47, and the Lim-domain proteins LMO2 and LDB1 (for review see [18]). The transcriptional master regulator TAL1 is a particular interesting candidate for *FUBP1* upstream regulation, because the protein plays an important role for maintenance of LT-HSCs [19] and for proper erythropoiesis [20], similarly to FUBP1 [11]. As a starting point to investigate a potential upstream regulation of *FUBP1* by TAL1, we quantified *FUBP1* and *TAL1* mRNA expression in several cell lines and primary cells by qPCR. As shown in **Fig 1B**, *FUBP1* is expressed in all established cell lines that we analysed and in human primary CD34^+^ haematopoietic progenitor cells. *TAL1* on the other hand is detectable in the chronic myelogenous leukaemia (CML) cell line K562 and in hCD34^+^ donor cells, while it is not expressed in the acute promyelocytic leukaemia cell line HL60 and in the human embryonic kidney cell line HEK293T (**Fig 1C**). Apparently, FUBP1 positive cells, which can be induced to undergo erythroid differentiation, express considerable *TAL1* levels. In these cells TAL1 could regulate *FUBP1* transcription.

**Fig 2:**
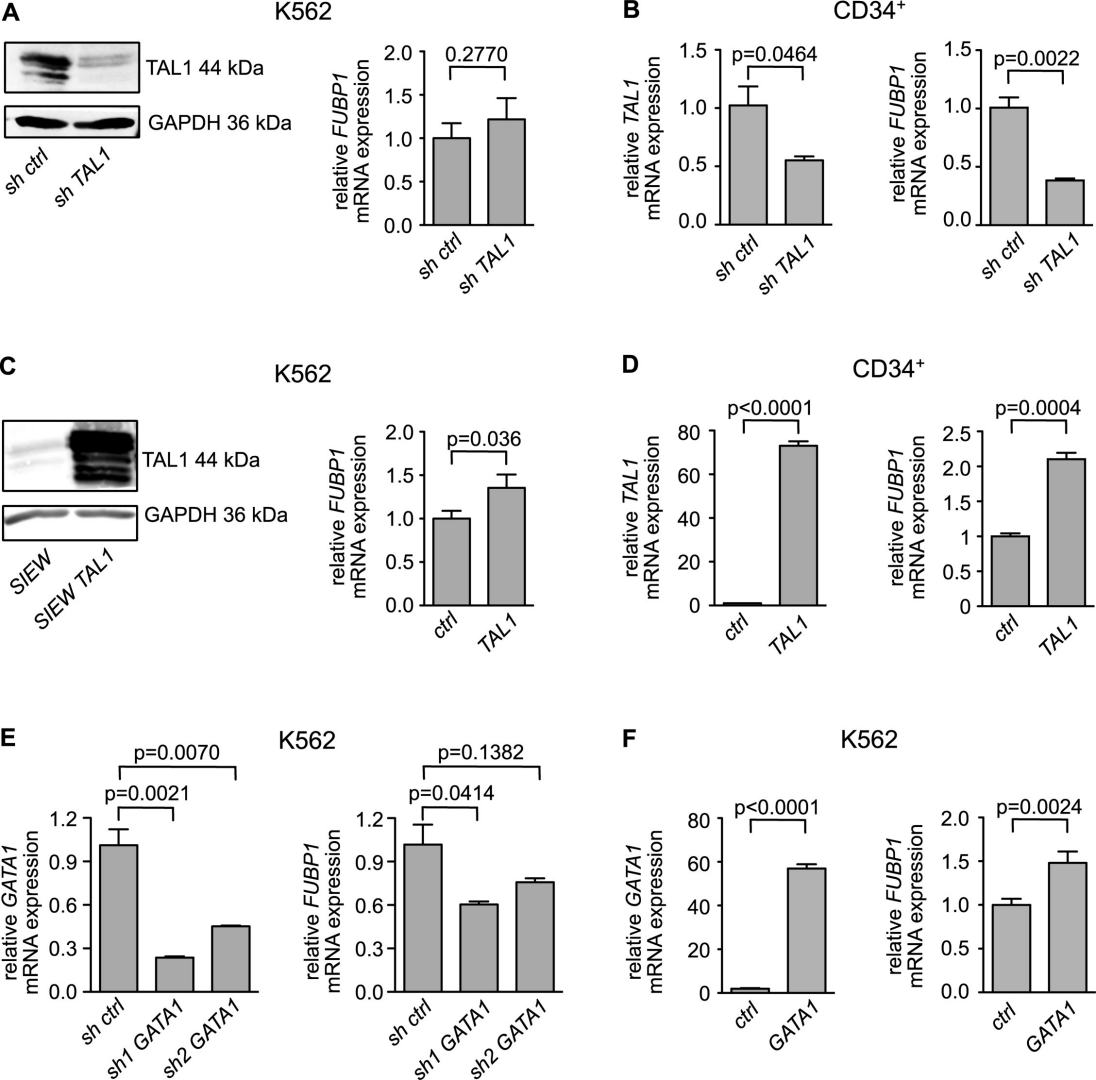
TAL1 expression correlates with *FUBP1* mRNA levels in the erythroleukemia cell line K562 and in primary human CD34^+^ blood stem/progenitor cells. **(A)** Knockdown of *TAL1* by shRNA in K562 cells (Western blot on the left side) has no effect on *FUBP1* mRNA expression (right). **(B)** Knockdown of *TAL1* in hCD34^+^ cells (left) results in diminished *FUBP1* expression (right). **(C)** Overexpression of *TAL1* in K562 cells (Western blot on the left side) leads to increased *FUBP1* expression levels. **(D)** Overexpression of *TAL1* in hCD34^+^ cells (left) results in increased *FUBP1* expression (right). **(E)** Knockdown of GATA1 by two different shRNAs in K562 cells reduces *FUBP1* mRNA expression levels. **(F)** Overexpression of GATA1 in K562 cells leads to increased *FUBP1* mRNA expression levels. Protein expression was detected by Western blotting, using GAPDH expression as loading control. mRNA expression levels were quantified by real-time PCR. Values were normalized to *GAPDH* expression and are presented as fold change relative to the vector control. Error bars display the mean results, with SD values calculated from three independent experiments.

We then analysed *FUBP1* and *TAL1* co-expression specifically during erythroid and megakaryocytic differentiation. For this purpose, we cultured primary human CD34^+^ cells for ten days in erythroid differentiation medium to induce erythropoiesis, or for ten days in megakaryocytic differentiation medium to achieve megakaryopoiesis (**S1 Fig**). Flow cytometry analysis of the cells for erythroid (CD235a/glycophorin A and CD71/transferrin receptor) and megakaryocytic (CD41/integrin α chain 2b (ITGA2B)) marker expression confirmed successful differentiation of the cells upon cytokine treatment (see **S2 Fig**). **Fig 1D** demonstrates that *FUBP1* and *TAL1* mRNA expression increased substantially during the erythroid differentiation process. While *FUBP1* expression remained stable, *TAL1* mRNA levels also rose during megakaryocytic differentiation on the mRNA level (**Fig 1E**). Subsequently, we analysed FUBP1 expression at the protein level and found that FUBP1 is increased during erythroid differentiation **(Fig 2F)** and transiently increased during megakaryopoiesis **(Fig 2G)**. The discrepancy between FUBP1 mRNA and protein levels in megakaryocytic differentiation warrants further examination.

Our examination shows that *FUBP1* and *TAL1* are co-expressed during erythroid differentiation of primary cells, and the increase of *FUBP1* expression in the differentiating cells correlates with rising *TAL1* levels. Thus, co-expression as a prerequisite for the regulation of *FUBP1* transcription by TAL1 is fulfilled.

### TAL1 expression regulates *FUBP1* mRNA levels

In the next step, we evaluated if TAL1 expression levels influence *FUBP1* transcription. For this purpose, we knocked down *TAL1* in K562 (**Fig 2A**, left) and in primary human CD34^+^ cells (**Fig 2B**, left). We then quantified the amount of *FUBP1* mRNA by real-time qPCR. Interestingly, *TAL1* knockdown had no effect on *FUBP1* expression in K562 cells but led to diminished *FUBP1* levels in primary hCD34^+^ cells (**Fig 2A** and **2B**, right panels). We then reversed the experimental set-up and over-expressed *TAL1* in K562 and hCD34^+^ cells (**Fig 2C** and **2D**, left). **Fig 2C** and **2D** right panels show that TAL1 overexpression results in a 1.4-fold upregulation of FUBP1 expression in K562 cells and in a 2.1-fold upregulation of FUBP1 mRNA levels in hCD34^+^ cell.

Because TAL1 and GATA1 often act together in gene expression regulation and the FUBP1 promoter displayed an E-box/GATA1-site combination, we analysed if GATA1 also influences FUBP1 expression. To this aim we knocked down GATA1 with two shRNAs, the knockdown led to a reduction in FUBP1 expression, which was dependent on the efficacy of the shRNA construct **(Fig 2E).** On the other hand, GATA1 over expression increased FUBP1 expression in K562 cells **(Fig 2F).** In conclusion our data strengthen the notion that TAL1 and GATA1 are upstream regulators of FUBP1.

### TAL1 binding to the *FUBP1* promoter region increases upon erythroid differentiation

**Fig 3:**
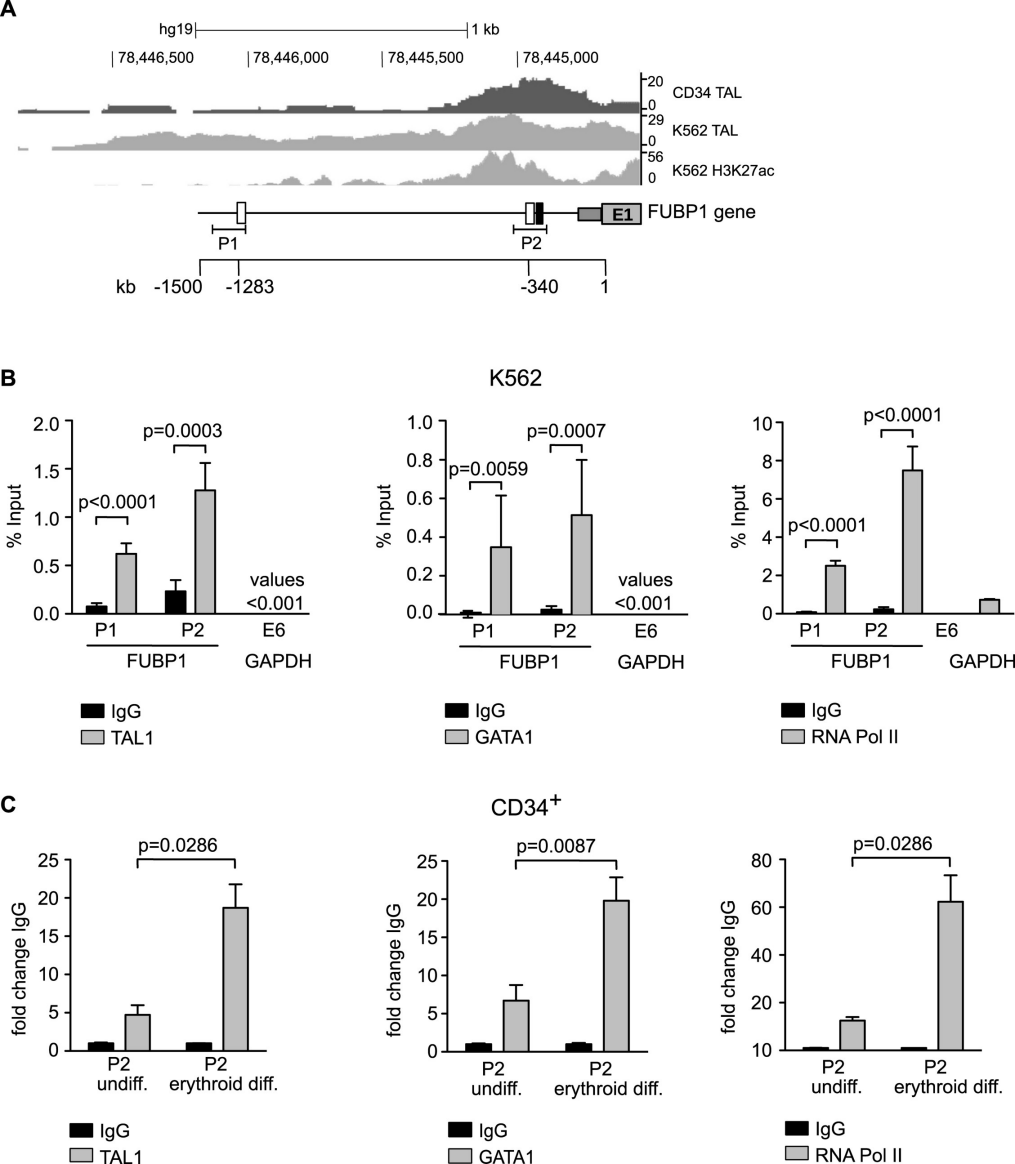
TAL1 binds to the *FUBP1* promoter region, and occupancy increases upon erythroid differentiation. **(A)** Binding of TAL1 to the *FUBP1* promoter and histone acetylation at the same locus. The promoter region of the *FUBP1* gene is shown (hg19, chromosome 1: 78,444,500), and included are the first exon of *FUBP1* (E1, grey box), the short 5’-UTR (dark grey box), E-boxes (white boxes) and GATA-motif (black box) in the promoter. The upper row of the panel shows binding of TAL1 to the *FUBP1* promoter in CD34^+^ human primary cells. The middle row displays binding of TAL1 to the *FUBP1* promoter region in K562 cells, and the lower row shows histone 3 acetylation (H3K27ac) in the *FUBP1* promoter region. The location of primer pairs P1 and P2 used for ChIP qPCR analysis is displayed. The scale bar at the bottom indicates the position of the E-boxes relative to the transcription start of the *FUBP1* gene. ChIP-sequencing data were taken from BloodChIP [21] and imported into the *UCSC-genome browser*. **(B)** ChIP analyses of TAL1, GATA1 and RNA-Pol II binding to the *FUBP1* promoter in K562 cells using specific antibodies against TAL1 (left), GATA1 (middle) and POLII (right). ChIP-qPCR with primer pairs at distinct regions of the FUBP1 promoter (P1 and P2) indicate TAL1, GATA1 and POLII binding in proximity to the transcriptional start site. In contrast, no binding of TAL1 and GATA1 is detected within exon 6 of GAPDH, which serves as control. **(C)** TAL1, GATA1 and POLII binding to P2 at the *FUBP1* promoter is significantly increased upon erythroid differentiation of hCD34^+^ cells, calculated as fold change of enrichment at P1 (% Input data are provided in **S3 Fig**). ChIPs were performed with an anti-TAL1 antibody (left), anti-GATA1 antibody (middle) and anti-POLII antibody (right). For ChIP IgG antibodies were used as an isotype-matched negative control. Error bars represent the mean results, with SD values derived from three independent experiments.

Publicly accessible chromatin immunoprecipitation sequencing (ChIP-Seq) data retrieved from the *BloodChIP* database [21] suggest binding of TAL1 at a region -530 bp to -170 bp upstream of the transcription start within the *FUBP1* promoter in K562 and hCD34^+^ cells. This region includes the E-box identified at -340 bp to -334 bp upstream to the transcription start and is also heavily H3K27-acetylated in K562 cells (**Fig 3A, P2**). However, the TAL1 peak at the transcriptional start site was relatively broad. Furthermore, no clear TAL1 ChIP-Seq peak was detected at an additional E-box -1283 to -1277 bp upstream of the transcription start (**Fig 3A**).

To confirm and further analyse a possible binding of TAL1 to the *FUBP1* promoter, we established our own TAL1 chromatin immunoprecipitation (ChIP) assay. **Fig 3A** schematically indicates the location of the two primer pairs P1 and P2 that were used for TAL1 and RNA polymerase 2 (POLII) ChIP experiments in K562 cells. The results displayed in **Fig 3B** (left) demonstrate the presence of TAL1 protein in the *FUBP1* promoter region. With both primer pairs P1 and P2, enrichment of *FUBP1* sequences could be detected upon anti-TAL1 chromatin precipitation compared to unspecific IgG precipitation. No enrichment of a sequence within exon 6 of GAPDH was observed when TAL1 was precipitated, confirming specificity of the binding of the transcription factor to the *FUBP1* promoter. As enrichment was higher at P2, the E-box/GATA motif -340 bp upstream of the *FUBP1* transcription start seems to be the most likely site to be occupied by TAL1. As shown in the right plot of **Fig 3B**, the same region assayed with primer pairs P1 and P2 produced a significant enrichment for POLII. As TAL1 has been reported to bind DNA in a complex with GATA1 at sequences containing juxtaposed E-box and GATA motifs, we also analysed binding of GATA1 to the *FUBP1* promoter. Anti-GATA1 chromatin precipitation resulted in significantly enriched *FUBP1* promoter sequences (**Fig 3B**, middle), similar to the TAL1- and POLII-ChIPs. No enrichment of TAL1, GATA1 and POLII was detected within exon 6 of GAPDH, or an intergenic region in chromosome 18 in K562 cells. This assay was used as an additional negative control (**S3 Fig**).

We then repeated our anti-TAL1 and anti-GATA1 ChIP experiments with primary hCD34^+^ cells that were incubated in erythroid differentiation medium. Both TAL1 and GATA1 were already detected at the *FUBP1* locus in undifferentiated hCD34^+^ cells. This binding of TAL1 and GATA1 to the *FUBP1* promoter increased significantly during erythropoiesis (**Fig 3C**, **S3 Fig**). Increased TAL1/GATA1 binding upon erythroid differentiation correlated with enhanced POLII recruitment to the *FUBP1* promoter (**Fig 3C,** right) and enhanced *FUBP1* transcription as shown before in **Fig 1D** and **S3 Fig**) demonstrates the specificity of the anti-TAL1, anti-GATA1 and anti-POLII antibodies in erythroid-differentiated primary hCD34^+^ cells, again using chromosome 18 sequences as a negative control for antibody binding.

Our results demonstrate the presence of TAL1 and GATA1 near the *FUBP1* transcription start. Recruitment of TAL1/GATA1 to the *FUBP1* promoter is increased upon erythroid differentiation, which correlates with augmented *FUBP1* transcription.

### The *FUBP1* promoter is activated by TAL1 in the context of an intact GATA binding site

**Fig 4:**
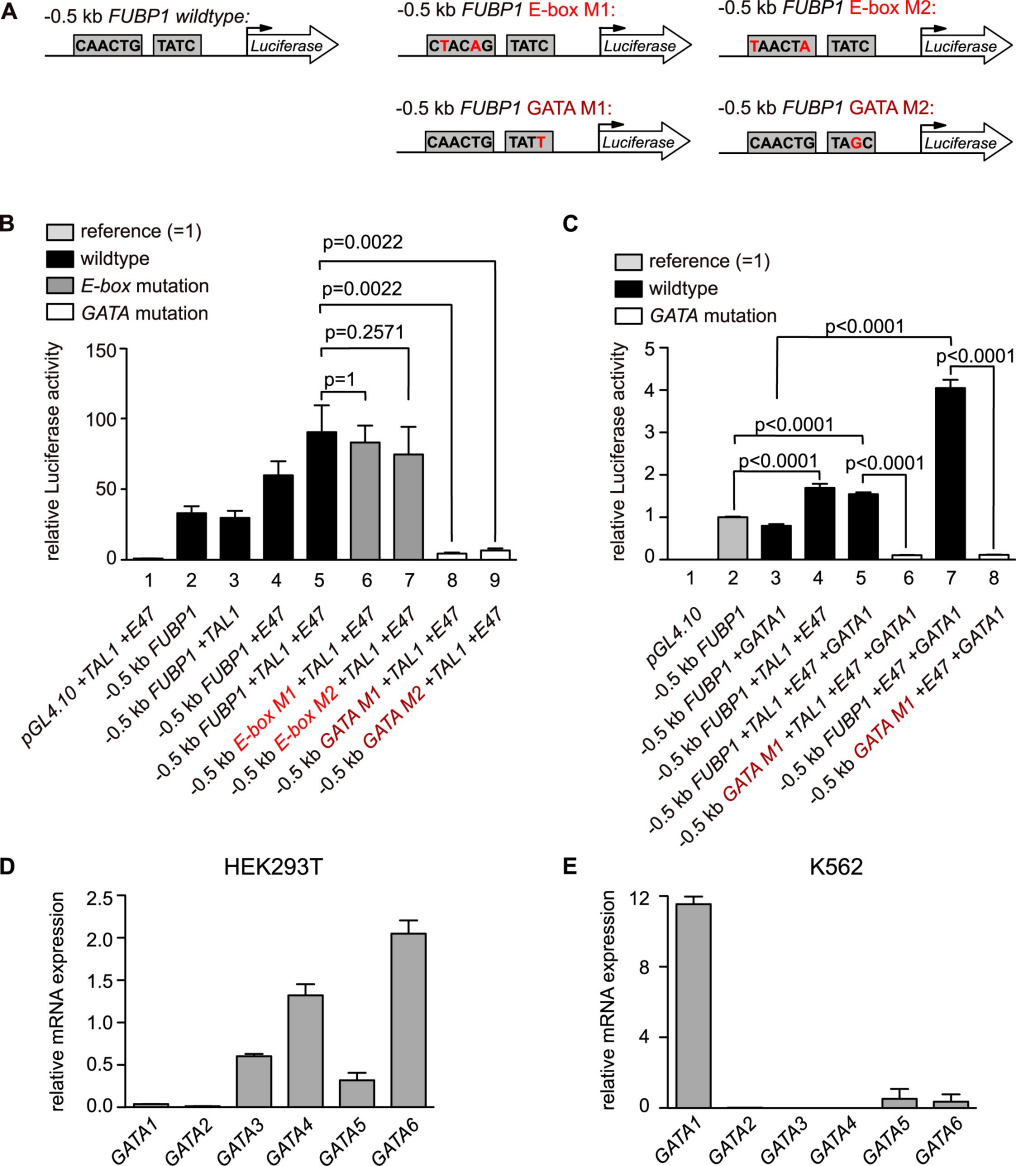
TAL1 associates with the *FUBP1* promoter via the *GATA* element to activate *FUBP1* expression. **(A)** Schematic presentation of the reporter constructs used for luciferase assays. 0.5 kb genomic sequence upstream of the *FUBP1* transcription start were cloned upstream of the luciferase cDNA (left panel). Two different point mutations (M1 and M2) within the E-box at position -340 bp upstream of transcription start (mutations in M1 at -339 bp and -335 bp, mutations in M2 at -340 bp and -335 bp, middle panel) and in the GATA-motif (*TATC*) -329 bp upstream of transcription start (mutation in M1 at -329 bp and at -328 bp in M2, right panel) were introduced by PCR. **(B)** HEK293T cells were transfected with the luciferase reporter constructs shown in **(A)**. Strong luciferase activity was measured in cells with TAL1/E47 over-expression (black bar 5) but to a lesser degree in cells expressing none or only one of the two proteins (black bars 2, 3, 4). Mutations in the GATA-motif (white bars 8 (M1), 9 (M2)) but not in the E-box (grey bars 6 (M1), 7 (M2)) led to a significant decrease of luciferase activity (P values are indicated). Values for luciferase activities are presented relative to luciferase activity of cells transfected with luciferase reporter vector without the *FUBP1* promoter region and normalized to β-galactosidase expression. **(C)** HEK293T cells were transfected with the luciferase reporter constructs shown in **(A)**. Exogenous expression of GATA1 additionally to E47 and TAL1 did not further increase luciferase activity (black bar 5), but expression of GATA1 and E47 significantly increased luciferase activity (black bar 7) compared to cells expressing only GATA1 or no exogenous protein (black bars 2 and 3). Mutations within the GATA-motif diminished GATA-1 induced luciferase activity (white bars 6 and 8)). Values for luciferase activities are presented relative to luciferase activity of cells transfected with luciferase reporter vector with the 0.5 kb *FUBP1* promoter region and normalized to β-galactosidase expression. Label colours refer to the constructs shown in **(A)**, exogenous expression of TAL1, E47, GATA1 and their combinations are indicated. **(D), (E)** mRNA expression levels of GATA family members in HEK293T **(D)** and K562 **(E)** cells. mRNA expression levels were quantified by real-time PCR. Values were normalized to GAPDH expression and are presented as fold change relative to the vector control. Error bars display the mean results, with SD values calculated from three independent experiments.

Our mRNA expression analysis suggests upregulation of *FUBP1* transcription by TAL1 and GATA1. To confirm that TAL1 binding to the *FUBP1* promoter indeed causes a transcriptional activation of *FUBP1*, we performed a reporter gene assay. For this, we cloned a -0.5 kb *FUBP1* promoter fragment upstream of a *luciferase* reporter cDNA to quantify promoter activity via measurement of luciferase activity (**Fig 4A**). This promoter construct contained the E-box/GATA combination upstream of the *FUBP1* transcription start site and mutant versions, in which the E-box site or the GATA1 site was mutated (see **Fig 1A**).

The -0.5 kb *FUBP1* promoter construct was highly active when transfected into HEK293T cells compared to the activity of the empty vector **(Fig 4B).** Reporter gene activity was not activated by TAL1 coexpression and was slightly increased upon transfection of an E47 expression vector. Interestingly, the luciferase signal was strongly increased upon co-transfection of the -0.5 kb *FUBP1* promoter construct with an expression vector for TAL1 together with its heterodimerisation partner E47 (**Fig 4B**). Strikingly, the mutation of the E-box did not influence activation by TAL1/E47, but mutation of the GATA motif (*TATC*) completely diminished reporter gene activity (**Fig 4B,** white bars). We speculate that this is partly, because endogenous GATA-factors present in HEK293T cells take part in the complex **(Fig 4D and 4E).** This notion is also supported by the fact that overexpression of TAL1 in HEK293 cells activates endogenous FUBP1 expression **(S4 Fig)**.

These results suggest that E47 and GATA1 might take part in promoter regulation. Thus we performed further reporter gene assays with GATA1. GATA1 alone did not activate the FUBP1 promoter **(Fig 4C)**. However, cotransfection of E47 with GATA1 strongly activated the promoter, this activation was stronger than the activation by E47/TAL1/GATA1. Mutation of the GATA1 site diminished activation by E47/GATA1 **(Fig 4C).** As in K562 cells GATA1 is the GATA factor with the strongest expression **(Fig 4D and 4E),** GATA1 might be part of the E47/TAL1 complex in these cells.

These data strongly suggest a functional role of the TAL1/E47 heterodimer with GATA1 in the transcriptional upregulation of *FUBP1*. Furthermore, this activity strongly depends on the GATA site at position -329 of the *FUBP1* promoter. Because TAL1, E47 and different GATA factors are present in distinct cell types to a varying amount, FUBP1 regulation might also be cell-type dependent.

### Knockdown of *FUBP1* in primary hCD34^+^ cells reduces the frequency of GYPA-positive cells during erythroid differentiation and diminishes erythroid colony formation

**Fig 5:**
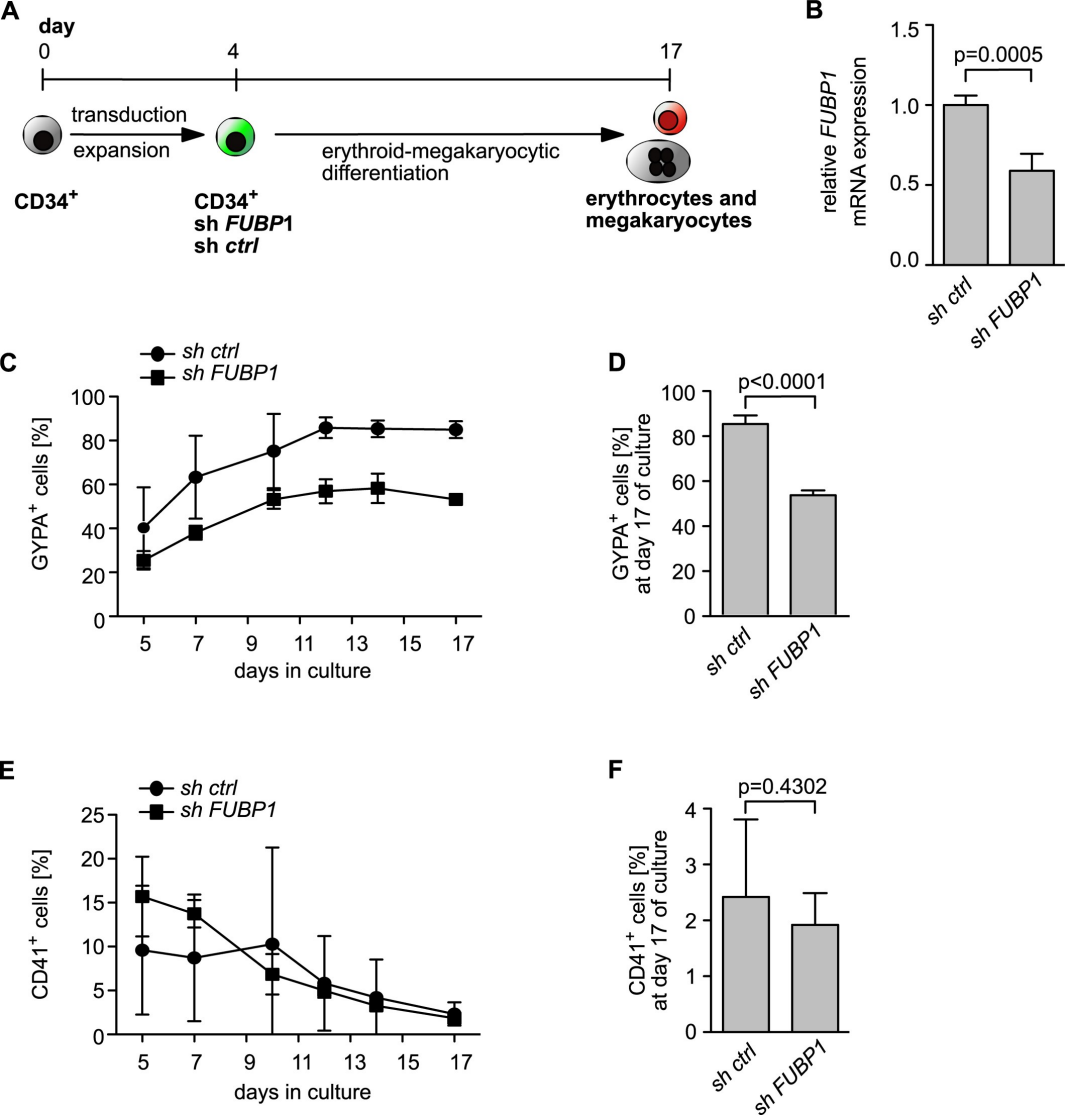
FUBP1 is important for efficient erythroid differentiation. **(A)** Schematic presentation of the experimental procedure used to analyse erythroid and megakaryocytic differentiation of primary hCD34^+^ cells, the composition of the differentiation medium is given in **S1 Fig. (B)** The efficiency of the shRNA-mediated *FUBP1* knockdown in hCD34^+^ primary cells was tested at the beginning (day zero) and at the end (day 17) of the experiment, bars represent the mean with SD of values detected at both time points. **(C-F).** The *FUBP1* knockdown led to reduced percentages of GYPA-positive cells during erythroid/megakaryocytic differentiation starting at day five of culture (**C, D**) but did not affect the percentage of CD41-positive cells (**E, F**). Bars in **(D)** and **(F)** represent the mean with SD of values obtained at day seventeen of culture.

**Fig 6:**
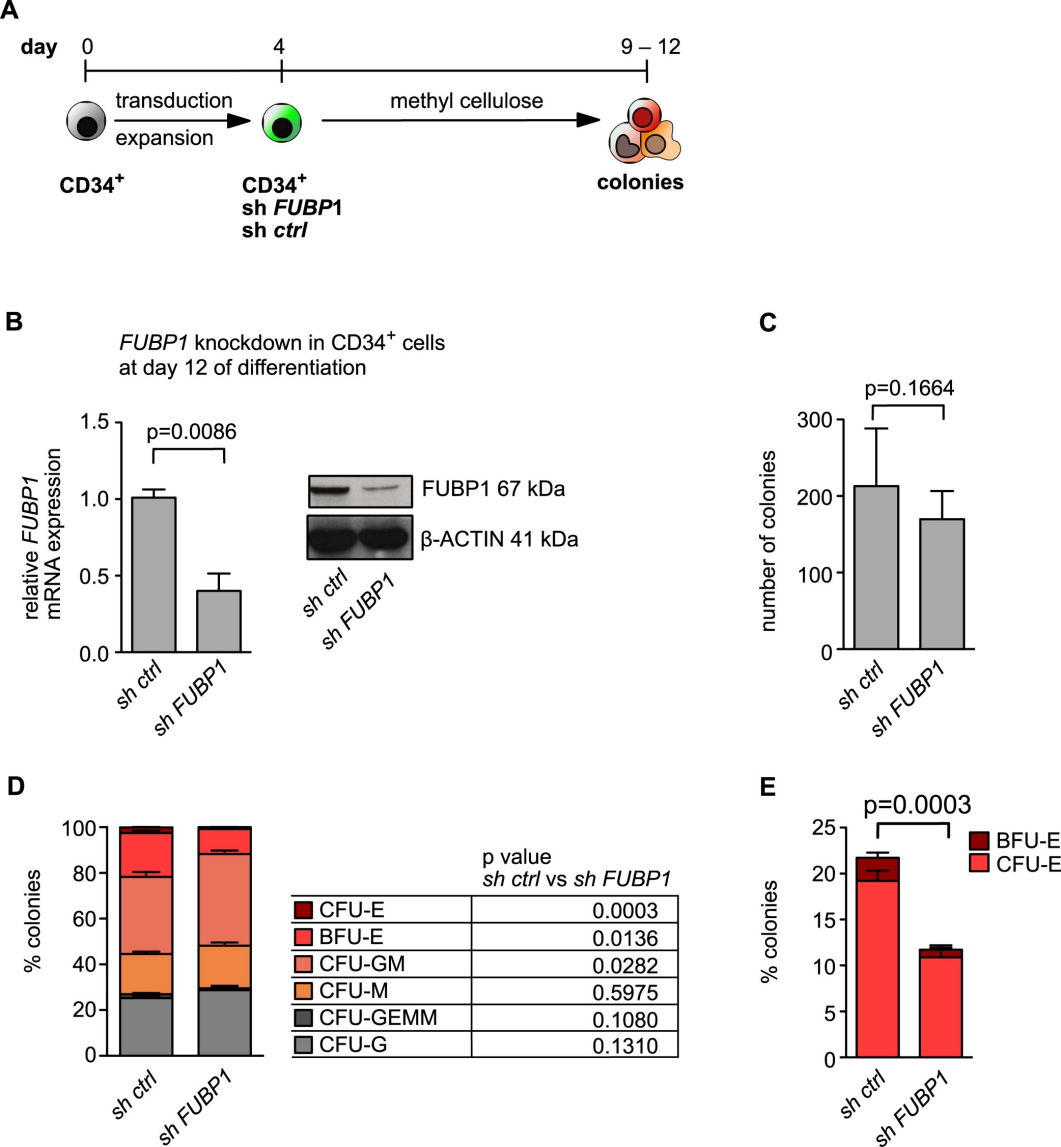
FUBP1 deficiency interferes with erythroid colony formation. **(A)** Schematic presentation of the experimental procedure chosen for the colony formation assay with human CD34^+^ cells shown in (**C-E)**, the composition of the differentiation medium is shown in **S1 Fig**. **(B)** The efficiency of the *FUBP1* knockdown in colony cells at the end of the experiment was verified on mRNA and protein level. β-ACTIN was detected as loading control. See also **S5 Fig** for full blot. mRNA expression was measured by quantitative real-time PCR. Values were normalized to *GAPDH* expression and are presented as fold change relative to the vector control. Error bars represent the mean results, with SD values derived from three independent experiments. **(C)** The total number of colonies derived from hCD34^+^ cells that were seeded in methylcellulose was not significantly reduced after *FUBP1* knockdown (p = 0.3531) compared to cells transduced with a control shRNA. **(D)** Colonies belonging to all hematopoietic lineages were formed by both CD34^+^ *FUBP1* knockdown and control cells after 9–12 days of culture in methylcellulose (CFU: colony forming unit, G: granulocyte, GEMM: granulocyte erythrocyte monocyte megakaryocyte, M: monocyte, GM: granulocyte-monocyte, E: erythrocyte, BFU: burst forming unit). **(E)** The number of erythroid colonies (CFU-E and BFU-E) was significantly reduced in *FUBP1* knockdown colony cells compared to cells with *FUBP1* wildtype levels (p = 0.0003).

To investigate the functional relevance of FUBP1 for erythroid differentiation of primary hCD34^+^ donor cells, we analysed the consequences of *FUBP1* knockdown on differentiation and colony formation. The experimental setup is presented in **Figs 5A** and **6A**. We transduced hCD34^+^ cells derived from two different donors with shRNA against *FUBP1* or control shRNA and confirmed the knockdown by real-time qPCR after 4 days in culture and sorting of successfully transduced cells (**Fig 5A and 5B**). Sorted cells were cultured for further 13 days in erythroid/megakaryocytic differentiation medium (**S1 Fig)** that allowed both erythroid and megakaryocytic differentiation of the cells. During this time, we quantified both erythrocyte (GYPA) and megakaryocytic (CD41) marker expression by flow cytometry. *FUBP1* mRNA expression analysis at the beginning and the end of the experiment revealed that the *FUBP1* knockdown was stable throughout the experiment (**Fig 5B**). When we analysed the cells after 12 days of differentiation for the proportion of GYPA-expressing erythroid cells, only 55% of the *FUBP1* knockdown cell population expressed GYPA as compared to 85% of GYPA-positive cells in the control shRNA-transduced cell population, and this difference remained stable until day thirteen of differentiation (**Fig 5C and 5D**). Apparently, reduced levels of FUBP1 expression led to a severe disturbance of erythroid differentiation. However, by comparing *FUBP1* knockdown cells with control shRNA-transduced cells we demonstrated that differentiation into megakaryocytes, as measured by the proportion of CD41-positive cells, was not affected by FUBP1 deficiency (**Fig 5E and 5F**).

We then went on to perform colony assays in methylcellulose with the shRNA-transduced hCD34^+^ cells that were derived from two different donors. Following transduction with shRNA against *FUBP1* or control shRNA and culturing for 4 days, GFP-positive (transduced) cells were FACS-sorted and cultured for 9 to 12 days in methyl cellulose under cytokine conditions that allowed growth of colonies derived from different haematopoietic lineages (**Fig 6A**). The knockdown of *FUBP1* was confirmed by qPCR and Western blotting (**Fig 6B**). Using a light microscope, colonies that consisted of granulocytes, monocytes/macrophages, erythrocytes and their mixed populations were identified and quantified. As a first result, we observed no significant difference in the total number of all colonies derived from *shFUBP1* or control shRNA-transduced cells (p = 0.3531, **Fig 6C**). While all types of colonies were formed by *FUBP1* knockdown and control cells, a prominent difference was seen with erythroid colonies (p = 0.0003). For both, early (BFU-E (burst forming units)) and more mature (CFU-E (colony forming units)) erythroid progenitor cells, only half the number of colonies grew from *shFUBP1* transduced hCD34^+^ cells compared to control shRNA-transduced cells (**Fig 6D and 6E**). These results support the conclusion that FUBP1 fulfils an important function during erythropoiesis.

## Discussion

During haematopoiesis, cell type-specific gene expression is established as cells differentiate from haematopoietic stem cells to adult functional cells. This process is controlled by the activity of lineage-specific transcription factors, which render the chromatin permissive or non-permissive for transcription. This leads to the establishment of defined chromatin patterns and consequently to stable cell type-specific gene expression [22].

The haematopoietic transcription factor TAL1 (T-cell acute lymphoblastic leukaemia) exerts an important function in embryonic HSC development and also at the megakaryocytic/erythroid branching in the adult organism [23]. During erythropoiesis, TAL1 activates genes, which are important for the specific function of the erythroid cells [24]. Here we show that TAL1 and GATA1 are direct regulators of the transcriptional regulator FUBP1 (*FUSE* binding protein 1). TAL1 contributes to increased *FUBP1* expression upon erythroid differentiation of primary hCD34+ progenitor/stem cells (**Figs 1 and 2**). FUBP1 in turn regulates transcription of several target genes, which are required for successful erythropoiesis.

FUBP1 binds to AT-rich *FUSE* DNA elements in open chromatin and is able to melt the DNA double-strand into single-stranded DNA structures. This way, FUBP1 contributes to the regulation of target genes such as *c-MYC*, *BIK*, *p21* and *USP29* [25]. However, little was known about how FUBP1 itself is regulated in context of differentiation. Our recent observation that FUBP1 is involved in erythroid differentiation [11, 12] prompted us to examine the involvement of erythropoiesis-associated transcription factors in transcriptional regulation of FUBP1 expression. Indeed, we found that TAL1 is a direct positive regulator of FUBP1 expression. The peak of TAL1 binding to the genomic *FUBP1* promoter sequence is at an E-box motif with the sequence CAACTG (**Figs 1A and 4A**), which could represent a TAL1 binding site. However, recently, the sequence CAGCTG has been suggested as the most common binding site for TAL1 [20]. Interestingly, we found that a GATA1 binding site adjacent to the E-box contributes strongly to TAL1 recruitment (**Fig 4**) and that GATA1 also occupies the *FUBP1* promoter (**Fig 3**). In contrast to TAL1, GATA1 expression is almost absent in haematopoietic stem and progenitor cells, but a strong upregulation of GATA1 expression is observed at the moment of erythroid and megakaryocytic commitment [26]. Accordingly, both TAL1 and GATA1 play an important role in the control of erythroid and megakaryocytic genes [20, 27]. At these sites, TAL1 and GATA1 act within a dynamic transcription factor network with other transcription factors and their cofactors on target genes. Concomitant binding of TAL1 and GATA1 often occurs at genomic DNA sites associated with erythroid genes, which they activate [20, 28, 29]. Remarkably, TAL1 can fulfil many functions in the absence of an intact DNA binding domain [23]. On a particular subset of target genes, TAL1 might also be recruited by GATA1 [30]. Our data suggest that the E-box found within the *FUBP1* promoter sequence acts as an anchoring point in case the TAL1 complex is associated at the GATA-site.

Our findings support the idea that cell type-specific gene expression is determined in sequential steps, which involves early priming of chromatin regions and the execution of cell type-restricted expression programs later during differentiation. Early in haematopoiesis, transcription factors such as RUNX1 mark genes for activation later during differentiation by establishing regions of open chromatin structure [22]. Interestingly, TAL1 binding to DNA predominantly occurs at such sites with an open chromatin structure [30]. As a consequence, both TAL1 and RUNX1 are found at sites belonging to erythroid and megakaryocytic genes in MEPs, where they may contribute to a bivalent chromatin status [31–33]. In erythroblasts, TAL1 can be recruited to genes by association with GATA1, inducing expression of lineage-specific genes [30]. Thus, the activation of *FUBP1* expression by TAL1 in the presence of GATA1 can be viewed as part of an erythroid molecular switch. As FUBP1 acts as a transcriptional regulator by itself that binds to open chromatin established by factors such as TAL1 and RUNX1, structural chromatin information is translated into erythroid gene activation.

Our observation that TAL1, GATA1 and FUBP1 are interconnected, is in part reflected by common functions during haematopoiesis. TAL1 and FUBP1 are important for haematopoietic stem cell maintenance, with TAL1 acting already at the haemangioblast state, when HSCs are established during embryonic haematopoiesis [34, 35]. In FUBP1 deficient mice HSCs are produced, but they display defects in HSC self-renewal [11]. TAL1-deficient mice die at day E9.5 during embryonic development, whereas *FUBP1*-deficient mice die at E15.5. TAL1, GATA1 and FUBP1 are important for normal erythropoiesis. The knockout of *Tal1* in adult mice results in anaemia, with a defect in late erythroid maturation [36]. *Gata1* knockout mice die between embryonic day 10.5 and 11.5 because of anaemia, as erythroid differentiation is blocked at the pro-erythroblast stage [37]. *Fubp1* knockout embryos are strongly anaemic, and maturation of haematopoietic progenitors (from murine embryonic stem (ES) cells) to the erythroid lineage is impaired as a consequence of *Fubp1* knockout in ES cells [11, 12]. Here, we demonstrate that reduced FUBP1 levels result in impaired erythroid differentiation of human CD34^+^ cells (**Figs 5 and 6**). Our data, according to which TAL1 and GATA1 regulate *FUBP1*, support a model in which TAL1 and GATA1 activate *FUBP1* expression during erythropoiesis. However, GATA1 and E47 might also have a role in *FUBP1* regulation. The interdependent connection between TAL1, E47 and GATA1 at the *FUBP1* promoter in distinct cell types warrants further examination.

Our data strengthen the notion that a TAL1/GATA1 complex not only regulates transcription of target genes with a direct erythroid function. Both proteins also act upstream of erythroid regulators such as the master regulator of erythropoiesis KLF1 [38]. In this work, we demonstrate that TAL1 is a direct transcriptional activator of *FUBP1* during erythropoiesis. FUBP1 in turn is a regulator of several genes with a functional role in erythropoiesis, among them *c-MYC*, *cyclin D2*, *p21* (*CDKN1A*) and *c-KIT*. Upregulation of these cell cycle regulators, or downregulation in case of cell cycle inhibitor *p21*, is required to allow a sufficient expansion and survival of erythroid progenitors [39]. This suggests an involvement of FUBP1 in apoptosis and proliferation control in erythroblasts. Indeed, *FUBP1* expression has been reported to parallel *c-MYC* expression, especially in highly proliferative cells, which are not finally differentiated [40]. It would be interesting to test whether expression of *FUBP1* needs to be repressed for the proliferation stop inducing the final maturation of erythrocytes, as it has been shown for *c-MYC* and *c-KIT [41, 42]*. Besides, transcriptional regulation by FUBP1 could play a role in erythroid differentiation beyond proliferation control. Its most prominent target gene c-MYC was found to be one of the minimal factors required for direct lineage conversion to erythroid cells in cooperation with TAL1, GATA1 and LMO2 [8]. Furthermore, c-MYC impacts erythroid heme synthesis [43, 44]. Thus, by regulating the expression of *c-MYC* and further target genes FUBP1 might be required for erythroid-specific cell functions.

Our observation that TAL1 and FUBP1 are part of a transcriptional network could have profound implications afar from erythropoiesis. It is well known that both factors are involved in several cancer types. Thus, it is well worth to examine if TAL1 or FUBP1 contribute to aberrant proliferation and differentiation in cancers, in which no obvious alterations in the loci of cell cycle and differentiation regulators themselves can be detected.

## Materials and methods

### Cell culture

K562 (ATCC no. CCL-243) and HL-60 (ATCC no. CCL-240) cells were cultured in RPMI 1640 and HEK293T cells (ATCC no. CRL-3216) in DMEM medium, both supplemented with 10% FCS, 2 mM Glutamine (*GIBCO*) and 1% Penicillin/Streptomycin (*PAA Laboratories*) at 37°C in a 5% CO_2_ atmosphere.

Peripheral blood from G-CSF-treated healthy donors was provided by the DRK Blutspendedienst Frankfurt. For enrichment of hCD34^+^ cells, mononuclear cells were isolated by density gradient centrifugation using *Ficoll-Paque* (*GE Healthcare*) and by positive immunomagnetical selection according to the manufacturer´s instructions (*CD34 MicroBead Kit*, *human*, *Miltenyi Biotec*). hCD34^+^ cells were expanded under serum-free conditions using Stem Span (*SFEM I*, *Stemcell Technologies*) supplemented with 100 ng/ml Flt3-LG, 20 ng/ml IL-3, 20 ng/ml IL-6 and 100 ng/ml SCF (all *Miltenyi*).

To induce erythroid differentiation, hCD34^+^ cells were cultured in erythroid differentiation medium consisting of *SFEM II* (*Stemcell Technologies*) supplemented with 1 U/ml erythropoietin (EPO) (*Roche*), 5 ng/ml IL-3, 20 ng/ml SCF, 2μM Dexamethason (*Sigma*), 0.2 μM estradiol (*Sigma*), and 100 U/ml penicillin/streptomycin. For megakaryocytic differentiation, hCD34^+^ cells were cultured in megakaryocytic differentiation medium consisting of *SFEM II* (*Stemcell Technologies*), supplemented with 30 ng/ml thrombopoietin (TPO) (*Miltenyi*), 7.5 ng/ml IL-6, 13.5 ng/ml IL-9 (*Miltenyi*), 1 ng/ml SCF and 100 u/ml penicillin/streptomycin for megakaryocytic differentiation.

For erythroid-megakaryocytic differentiation in liquid culture, isolated hCD34+ cells were maintained in serum-free expansion medium *SFEMII* (*Stemcell Technologies*) supplemented with 100 ng/ml SCF, 10 ng/ml IL-3, 10 ng/ml IL-6, 0.5 U/ml EPO and 50 ng/ml TPO. Human primary cells from healthy volunteers were gathered with written informed consent and approval by the Ethics Committee of the Goethe University Frankfurt (permit #329–10). All experiments were performed in accordance with relevant guidelines and regulations. An overview of the different differentiation media is shown in **S1 Fig**.

### Knockdown and overexpression of FUBP1, TAL1 and GATA1

Knockdown of *FUBP1*, *TAL1* and *GATA1* was performed by lentiviral transduction. The following shRNAs were used:

*sh TAL1*: 5´-ACCTCGACAAGAAGCTCAGCAAGAATTCAAGAGATTCTTGCTGAGCTTCTTGTCTTTTT-3´

*sh ctrl*: 5´-GATCCCCTAACGACGCGACGACGTAATTCAAGAGATTACGTCGTCGCGTCGTTATTTTT-3´

*sh1 FUBP1*: 5´-GATCCCCGGCAGGAACGGATCCAAATTTCAAGAGAATTTGGATCCGTTCCTGCCTTTTT-3´

*sh2 FUBP1*: 5´-GATCCCCGATTACAGGAGACCCATATAATTCAAGAGATTATATGGGTCTCCTGTAATCTTTTT-3´

*sh1 GATA1*: 5´-TGCTGTTGACAGTGAGCGCCCTCATCTTGTGATAGAGGCCTGAAGCCACAGATGGCCTCTATCACAAGATGAGGATGCCTACTGCCTCGG-3´

sh2 GATA1: 5´-TGCTGTTGACAGTGAGCGCCCAGCTTGTAGTAGAGGCCGCTGAAGCCACAGATGCGGCCTCTACTACAAGCTGGATGCCTACTGCCTCGG-3´

For production of lentiviral particles, 1.4x10^7^ HEK293T cells were seeded in T175 cell culture flasks and transfected on the following day with 28.8 μg shRNA vector, 10.1 μg pMD2.G packaging plasmid, 18.7 μg pCMVdelta8.91 packaging plasmid and 144 μL PEI (1mg/ml). The supernatant was harvested 24 and 48 h after transfection and stored at 4°C. For concentration of lentiviral particles, the supernatant was centrifuged at 400 x g for 5 min at 4°C and sterile filtered (PVDF, 0.45 μM). The supernatant was underlain with 5 mL of 20% sucrose and centrifuged for 20,000 x g for 2 h at 4°C. The lentiviral pellet was resuspended in 500 μl *SFEM I* and stored at -80°C.

For knockdown or overexpression analysis, 1.4x10^6^ K562 cells were seeded in 6-well plates with 1.5 ml RPMI and incubated for 4 h. Subsequently, 100 μl concentrated virus and 30 μl protamine sulfate (0.4 mg/ml) were added to the cells and centrifuged at 2,000 x g for 90 min at 32°C. The cells were incubated overnight. CD34^+^ cells were transduced as described above. 5.0x10^5^ cells were seeded in 24-well plates with 500 μl *SFEM I* and incubated for 24 h. 50 μl concentrated virus and 5 μl protamine sulphate were added to the cells and centrifuged at 1,400 x g for 60 min at 32°C.

### Quantitative real-time PCR

RNA was isolated using the *RNeasy Mini Kit* (*Qiagen*) according to the manufacturer´s instructions. For cDNA synthesis, 1.5 μg RNA were transcribed with the *Omniscript Reverse Transcription Kit* (*Qiagen*) according to the manufacturer´s manual with an additional “on column” digest. qPCR assays were performed with a *LightCycler480* (*Roche*) using the *SYBR Green Real-Time PCR Master Mix* (*life technologies*), and relative gene expression was calculated using the ΔΔC_T_ method [45]. Gene expression was normalized to the expression level of the housekeeping gene *GAPDH*, and the log2 fold change over the control sample was calculated.

DNA primers that were used for the individual qPCR analyses are listed in **S1 Table**.

### Western blot

For Western blots the samples were run on an acrylamide gel and blotted onto a membrane using standard techniques. Blots were cut into half and incubated with the indicated antibodies (**Fig 1** and **Fig 6**, see also **S5 Fig**. **Fig 2A** and **S5 Fig**). Loading controls are provided from the same blots. Primary antibodies and antibody dilutions used for detection of FUBP1 and TAL1 in immunoblots are listed in **S2 Table**. GAPDH expression was assessed as a loading control. HRP-linked donkey anti-rabbit antibody (*GE Healthcare*, NA934, 1:2000) and HRP-linked rabbit anti-goat antibody (*Invitrogen*, 81–1620, 1:10,000) were used as secondary antibodies. Full blots are shown in **S5 Fig**.

### Chromatin immunoprecipitation (ChIP) assay

ChIP assays were performed according to the *X-ChIP* protocol from *Abcam* (http://www.abcam.com/protocols/cross-linking-chromatin-immunoprecipitation-x-chip-protocol), with the following modifications: after formaldehyde fixation, cells were rinsed three times with 10 ml ice cold PBS and centrifuged at 1,200 rpm, 5 min, 4°C. The cell pellet was resuspended in 600 μl lysis buffer per 1x10^7^ cells. For immunoprecipitation, 3–5 μg of specific antibody and 25 μl blocked protein G beads were used (antibodies employed for ChIP analysis are listed in **S2 Table**). Immunoprecipitated samples were washed three times in low salt wash buffer and three times in high salt wash buffer. Precipitated DNA was isolated using the *ChIP DNA Clean and Concentrator Kit* (*Zymo Research*) according to the manufacturer´s manual and analysed by qPCR. DNA enrichment was calculated as percentage of the input. Bar graphs show the mean and standard deviation of at least two independent experiments, each performed in duplicates.

DNA oligonucleotides that were used as primers for ChIP PCR analyses are listed in **S1 Table**.

### Luciferase assay

The 0.5 kb genomic sequence upstream of the translation start codon *ATG* of the *FUBP1* promoter was amplified by PCR from human genomic DNA isolated from Hep3B cells and inserted into the *KpnI/HindIII* restriction sites of the *pGL4*.*10* luciferase reporter vector (*Promega*) upstream of the *luc2* gene. Point mutations in the E-box located at position -340 bp of the *FUBP1* promoter and in the juxtaposed *GATA* motif were introduced using the *Phusion Site-Directed Mutagenesis Kit* (*Thermo Fisher Scientific*) according to the manufacturer´s manual.

For promoter activity assays, 1x10^5^ HEK293T cells per well were seeded in 24-well plates. The following day, cells were transfected with 1 μg plasmid and 4 μl PEI per well. Two days after transfection cells were scraped off the plate, washed with ice-cold PBS at 2,400 rpm for 5 min, and the pellet was resuspended in 110 μl lysis buffer (50 mM Tris pH 7.4, 50 mM NaCl, 1% v/v Triton X-100). After incubation for 15 min on ice samples were centrifuged at 2,400 rpm for 5 minutes, and 10 μl of supernatant were transferred on a 96 well plate. 90 μl luciferase buffer were added (21.625 mM Glycyl-Glycine, 1 mM ATP, 10 mM MgSO_4_, 0.075 mM Luciferin) and light emission was measured using a *LUMIstar Galaxy* microplate reader (*BMG Labtech*). The quantified luciferase activity was normalized to transfection efficiency by calculating luciferase activity relative to co-transfected β-galactosidase activity. To measure β-galactosidase activity, 5 μl of supernatant and 95 μl of buffer (11.1 mM MgCl_2_, 50 mM β-Mercaptoethanol, 3.25 mM *o*-Nitrophenyl-β-D-galactopyranosid (ONPG), 74.4 mM sodium phosphate) were mixed in a 96-well plate, and absorption was measured at 420 nm after 10–20 minutes of incubation (*SPECTRAmax 340*, *Molecular Devices*). Bar graphs show the mean and standard deviation of three independent experiments, each measured in duplicates.

### Flow cytometry

To assess GYPA and CD41 cell surface expression, cells were washed with ice-cold PBS and stained with anti-human CD41a APC (*HIP8*; *eBioscience*) or anti-human CD235a APC (*HIR2*/*GA-R2*, *eBioscience*) antibody (**S2 Table**) according to the manufacturer´s instructions. Fluorescence was quantified using a *BD FACSCanto II* (*BD Biosciences*). The gating strategy and data are provided (**S2 Data and fcs files**)

### Colony formation assays

For colony formation assays, successfully transduced GFP-expressing cells were sorted. One day after sorting 1-2x10^3^ cells in 210 μl *SFEM I* medium (*Stemcell Technologies*) supplemented with 3% penicillin/streptomycin were mixed with 3 ml *MethoCult Classic Methylcellulose* (*Stemcell Technologies*) and plated in two 3 cm dishes (**S1 Fig**). After 9–12 days colonies were scored using a light microscope.

### Statistical analysis

Results performed in at least three replicates were statistically analysed using *GraphPad Prism* software. Unpaired and parametric two-tailed Student’s t-test was used to calculate statistical significance. The data underlying the graphs are provided in the Supplementary Information.

## Supporting information

S1 Fig Expansion and differentiation of donor-derived human CD34^+^ stem and progenitor cells. Donor-derived human CD34^+^ stem and progenitor cells were maintained in serum-free SFEM I medium supplemented with 100 ng/ml Flt3-LG, 20 ng/ml IL-3, 20 ng/ml IL-6 and 100 ng/ml SCF for expansion. To induce either megakaryocytic or erythroid differentiation or both, cells were cultured in the respective media: megakaryocytic differentiation medium: SFEM II supplemented with 30 ng/ml thrombopoietin (TPO), 7.5 ng/ml IL-6, 13.5 ng/ml IL-9, 1 ng/ml SCF and 100 u/ml Penicillin/Streptomycin (Pen/Strep); erythroid differentiation medium: SFEM II supplemented with 1 U/ml erythropoietin (EPO), 5 ng/ml IL-3, 20 ng/ml SCF, 2 μM Dexamethason, 0.2 μM estradiol and 100 U/ml PenStrep; erythroid/megakaryocytic differentiation medium: SFEM II supplemented with 100 ng/ml SCF, 10 ng/ml IL-3, 10 ng/ml IL-6, 0.5 U/ml EPO and 50 ng/ml TPO. To induce growth of colonies derived from different hematopoietic lineages, cells were resuspended in SFEM I medium supplemented with 3% Pen/Strep and mixed with 3 ml *MethoCult Classic Methylcellulose*.
(TIFF)

S2 Fig Lineage marker expression during erythroid and megakaryocytic differentiation of CD34^+^ human primary cells. Successful differentiation of human CD34^+^ primary cells was tested by mRNA quantification and cell surface expression of differentiation markers. **(A)** Cells that were cultured in erythroid differentiation medium showed an up-regulation of *CD71* and *GYPA* (*CD235a*) mRNA expression, tested at day 7 and 10 of differentiation. **(B)** At day 12 of erythroid differentiation, 94.4% of the cells were CD235a-APC-positive according to flow cytometry analysis. **(C)** Cells cultured in megakaryocytic differentiation medium showed an up-regulation of *CD41* mRNA expression at day 7 and 10 of differentiation. **(D)** Flow cytometry analysis revealed that 84.8% of the cells were CD41-positive at day 12 of megakaryocytic differentiation.
(TIFF)

S3 Fig ChIP analyses show enrichment of TAL1, GATA1 and POLII at the FUBP1 promoter and within unrelated DNA on chromosome 18. **(A)** ChIP results, depicted as % of the input, demonstrate increased binding of TAL1, GATA1 and POLII at P2 in hCD34^+^ cells upon erythroid differentiation. **(B)** Primer pair binding within an intergenic region of the chromosome 18 DNA sequence and amplifying a fragment from Chr18:65075058 to Chr18:65075181, genome version HG38, was used as a negative control for qPCR analysis following ChIP. The antibodies against TAL1, GATA1 and RNA Pol II showed no unspecific binding within this chromosome 18 region in K562 cells (left), undifferentiated human CD34^+^ primary cells or human CD34+ cells incubated for 12 days in erythroid differentiation medium (right). IgG was used as isotype-matched control. Error bars represent the mean results, with SD values derived from at least two independent experiments.
(TIFF)

S4 Fig Overexpression of TAL1 in HEK293T cells increases FUBP1 mRNA expression. Overexpression of *TAL1* in HEK293T cells (left) leads to increased *FUBP1* expression levels (right). mRNA expression levels were quantified by real-time PCR. Values were normalized to *GAPDH* expression and are presented as fold change relative to the vector control. Error bars display the mean results, with SD values calculated from three experiments.
(TIFF)

S5 Fig Extended Western blot presented in Figs 1 and 2 and 6. The uncropped Western blots are provided. **A**. Related to Fig 1F. **B**. Related to Fig 1G. **C**. Related to Fig 2A. **D.** Related to Fig 2C. **E**. Related to Fig 6B.
(EPS)

S1 Data Excel file with the data presented in the manuscript. The data points from which graphs and statistics have been calculated are provided.
(XLSX)

S2 Data FACS files, related to Fig 5 (Fig 5D and 5F) showing the CD41 and GYPA gating. (PDF)

S1 File Control 1, FACS fcs file, related to Fig 5D and 5F. Raw data shControl.
(FCS)

S2 File Control 2, FACS fcs file, related to Fig 5D and 5F. Raw data shControl.
(FCS)

S3 File Control 3, FACS fcs file, related to Fig 5D and 5F. Raw data shControl.
(FCS)

S4 File shFUBP1 1, FACS fcs file, related to Fig 5D and 5F. Raw data shFUBP1.
(FCS)

S5 File shFUBP1 2, FACS fcs file, related to Fig 5D and 5F. Raw data shFUBP1.
(FCS)

S6 File shFUBP1 2, FACS fcs file, related to Fig 5D and 5F. Raw data shFUBP1.
(FCS)

S1 Table Sequences of primers used for qPCRs. (DOCX)

S2 Table Primary antibodies used for protein detection in immunoblots. (DOCX)
