# Supplementary figures and images for: FUSE binding protein 1 (FUBP1) expression is upregulated by T-cell acute lymphocytic leukemia protein 1 (TAL1) and required for efficient erythroid differentiation
Source: PLoS One. 2019 Jan 17;14(1):e0210515. doi: 10.1371/journal.pone.0210515 (PMC6336336; doi:10.1371/journal.pone.0210515)

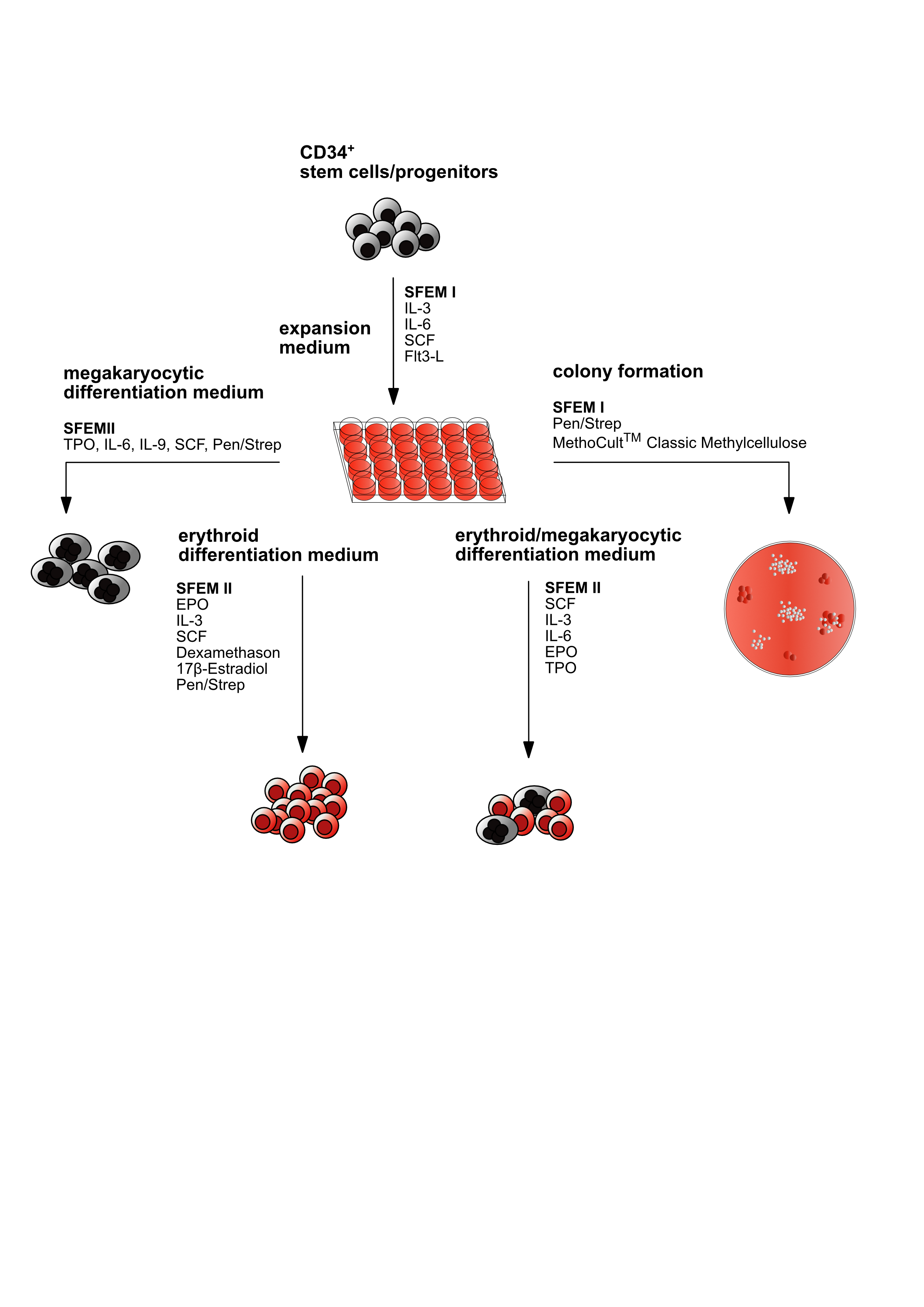

Supplement: S1 Fig — Donor-derived human CD34+ stem and progenitor cells were maintained in serum-free SFEM I medium supplemented with 100 ng/ml Flt3-LG, 20 ng/ml IL-3, 20 ng/ml IL-6 and 100 ng/ml SCF for expansion. To induce either megakaryocytic or erythroid differentiation or both, cells were cultured in the respective media: megakaryocytic differentiation medium: SFEM II supplemented with 30 ng/ml thrombopoietin (TPO), 7.5 ng/ml IL-6, 13.5 ng/ml IL-9, 1 ng/ml SCF and 100 u/ml Penicillin/Streptomycin (Pen/Strep); erythroid differentiation medium: SFEM II supplemented with 1 U/ml erythropoietin (EPO), 5 ng/ml IL-3, 20 ng/ml SCF, 2 μM Dexamethason, 0.2 μM estradiol and 100 U/ml PenStrep; erythroid/megakaryocytic differentiation medium: SFEM II supplemented with 100 ng/ml SCF, 10 ng/ml IL-3, 10 ng/ml IL-6, 0.5 U/ml EPO and 50 ng/ml TPO. To induce growth of colonies derived from different hematopoietic lineages, cells were resuspended in SFEM I medium supplemented with 3% Pen/Strep and mixed with 3 ml MethoCult Classic Methylcellulose. (TIFF) [file pone.0210515.s001.tiff]

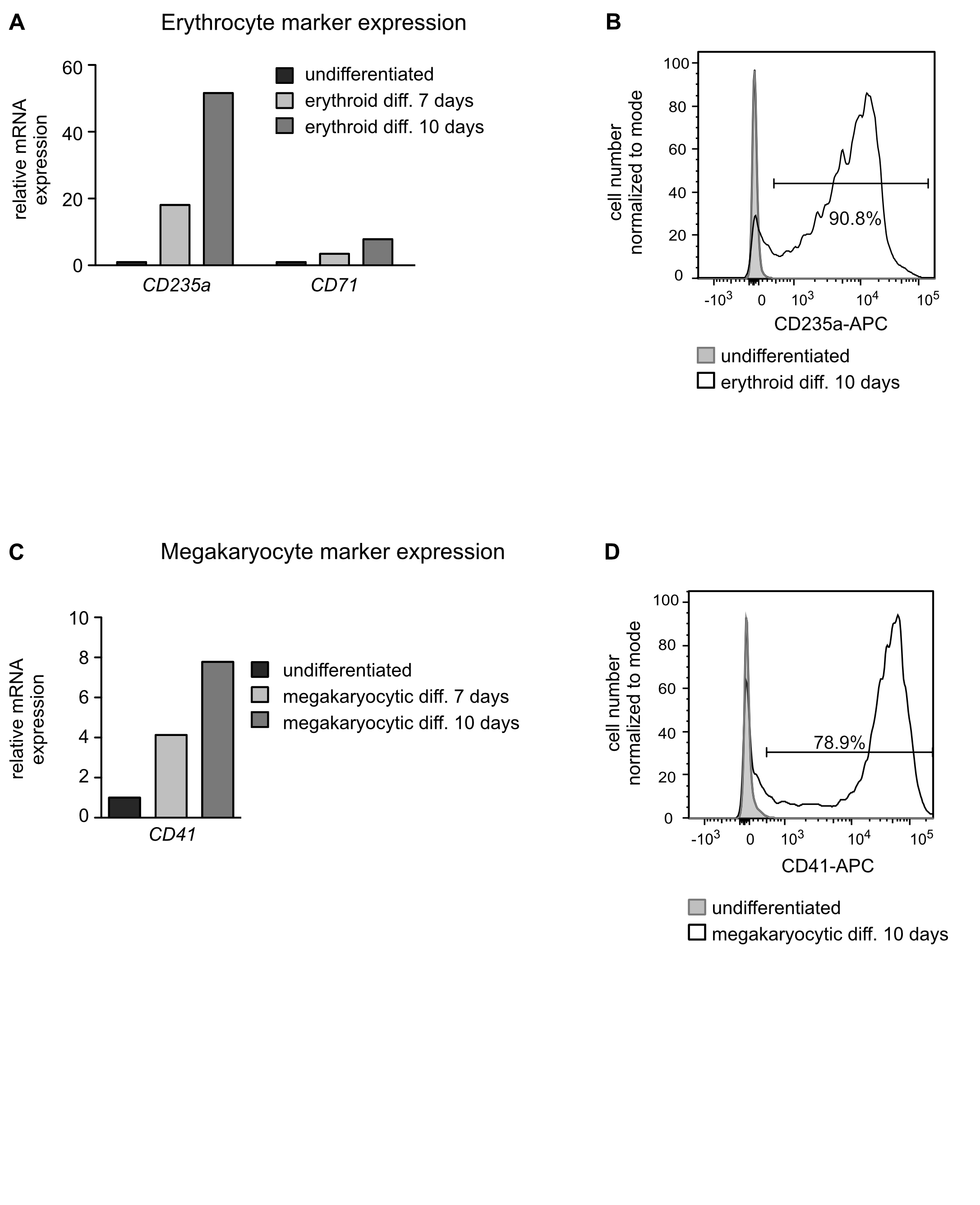

Supplement: S2 Fig — Successful differentiation of human CD34+ primary cells was tested by mRNA quantification and cell surface expression of differentiation markers. (A) Cells that were cultured in erythroid differentiation medium showed an up-regulation of CD71 and GYPA (CD235a) mRNA expression, tested at day 7 and 10 of differentiation. (B) At day 12 of erythroid differentiation, 94.4% of the cells were CD235a-APC-positive according to flow cytometry analysis. (C) Cells cultured in megakaryocytic differentiation medium showed an up-regulation of CD41 mRNA expression at day 7 and 10 of differentiation. (D) Flow cytometry analysis revealed that 84.8% of the cells were CD41-positive at day 12 of megakaryocytic differentiation. (TIFF) [file pone.0210515.s002.tiff]

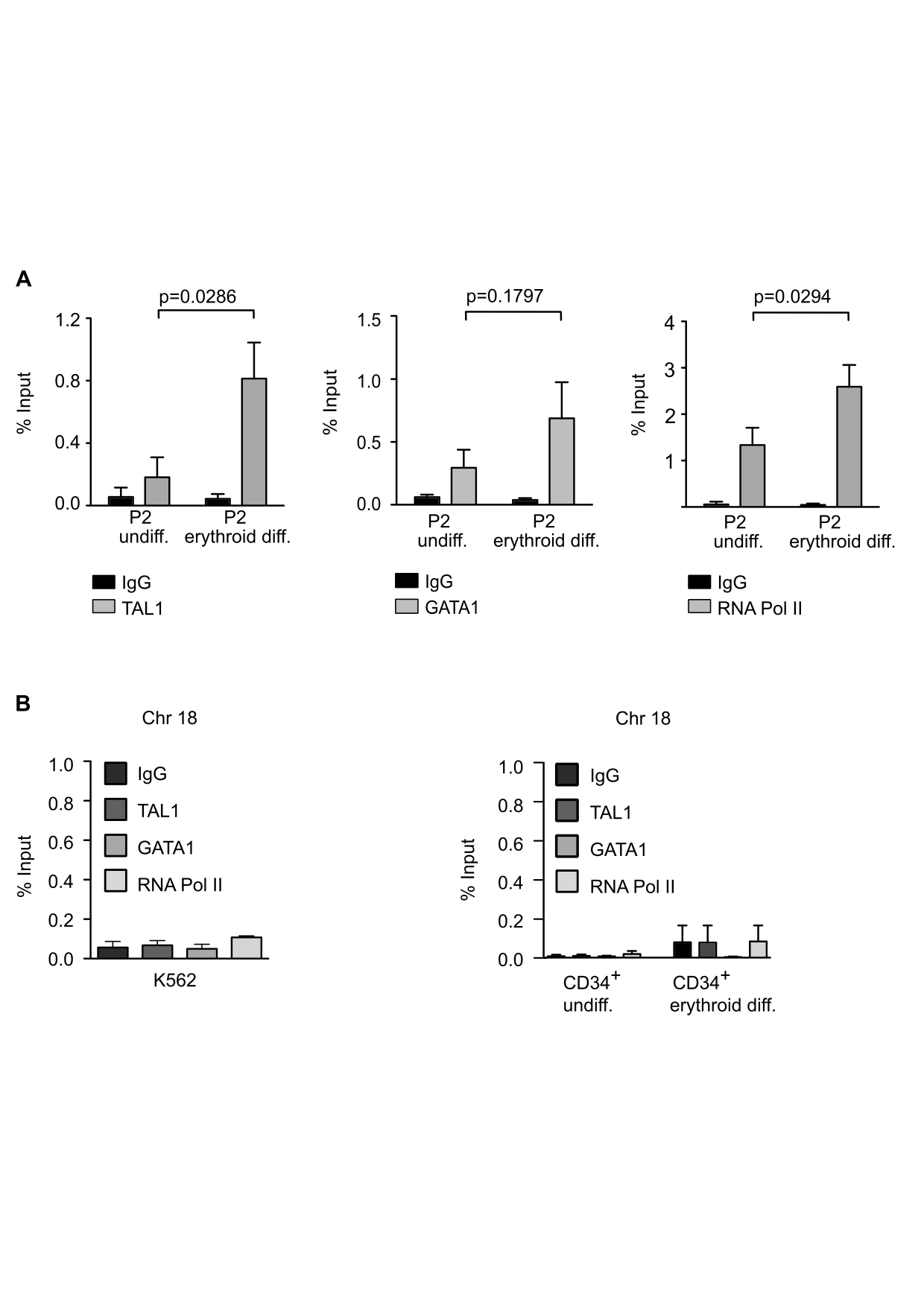

Supplement: S3 Fig — (A) ChIP results, depicted as % of the input, demonstrate increased binding of TAL1, GATA1 and POLII at P2 in hCD34+ cells upon erythroid differentiation. (B) Primer pair binding within an intergenic region of the chromosome 18 DNA sequence and amplifying a fragment from Chr18:65075058 to Chr18:65075181, genome version HG38, was used as a negative control for qPCR analysis following ChIP. The antibodies against TAL1, GATA1 and RNA Pol II showed no unspecific binding within this chromosome 18 region in K562 cells (left), undifferentiated human CD34+ primary cells or human CD34+ cells incubated for 12 days in erythroid differentiation medium (right). IgG was used as isotype-matched control. Error bars represent the mean results, with SD values derived from at least two independent experiments. (TIFF) [file pone.0210515.s003.tiff]

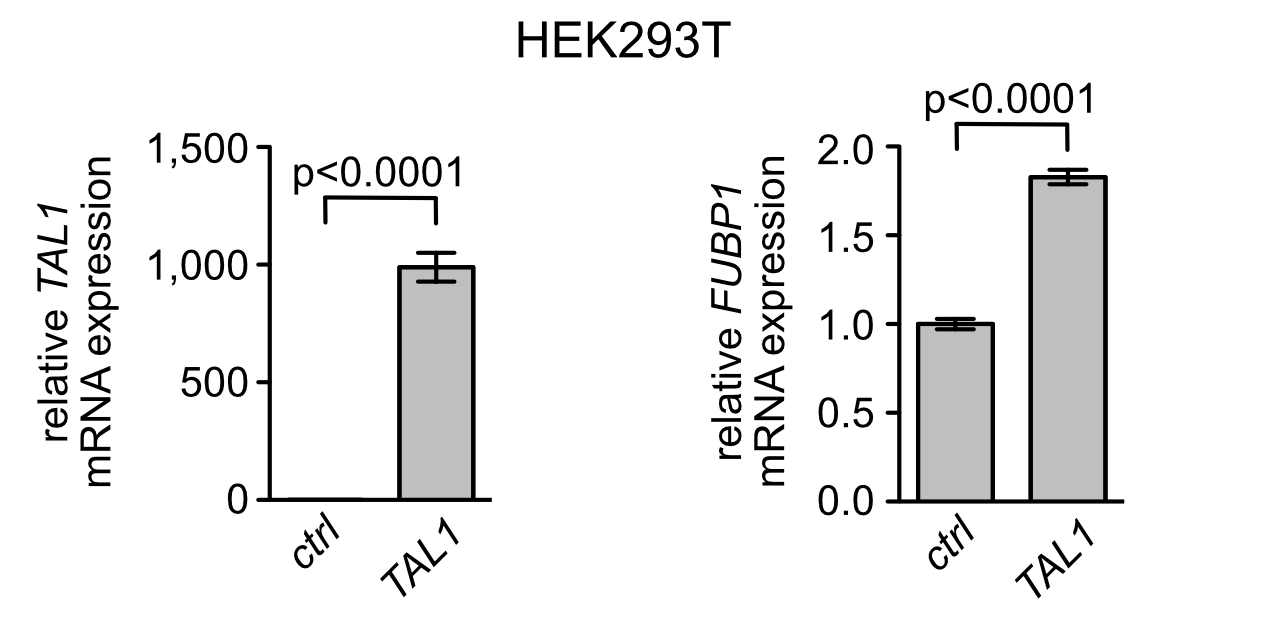

Supplement: S4 Fig — Overexpression of TAL1 in HEK293T cells (left) leads to increased FUBP1 expression levels (right). mRNA expression levels were quantified by real-time PCR. Values were normalized to GAPDH expression and are presented as fold change relative to the vector control. Error bars display the mean results, with SD values calculated from three experiments. (TIFF) [file pone.0210515.s004.tiff]
