# Supplementary material for: FUSE binding protein 1 (FUBP1) expression is upregulated by T-cell acute lymphocytic leukemia protein 1 (TAL1) and required for efficient erythroid differentiation
Source: PLoS One. 2019 Jan 17;14(1):e0210515. doi: 10.1371/journal.pone.0210515 (PMC6336336; doi:10.1371/journal.pone.0210515)
Supplement: S2 Data — (PDF) [file pone.0210515.s007.pdf]

Batch Analysis Report

Run Date: 10/5/15 11:05 AM   Experiment: 20151005 CD34 FUBP1 TAL1 EM Dif d17
User ID: JasminY
Statistics Output: D:\BDExport\Experiment\AG Lausen\Jasmin\20151005\CFb\Batch_Analysis_05102015110424.csv
Worksheet PDF Output: D:\BDExport\Experiment\AG

CD34

Tube	Status	Run Time	
wt ung	OK	10/5/15 11:05 AM	
SEW ung	OK	10/5/15 11:05 AM	
~~SEW E CD235 APC~~	~~OK~~	~~10/5/15 11:05 AM~~	unrelated experiments
~~SEW M CD41 PB~~	~~OK~~	~~10/5/15 11:05 AM~~	
SEW EM CD41 CD235a 1	OK	10/5/15 11:06 AM	shControl 1
SEW EM CD41 CD235a 2	OK	10/5/15 11:06 AM	shControl 2
SEW EM CD41 CD235a 3	OK	10/5/15 11:06 AM	shControl 3
FUBP1 EM CD41 CD235a 1	OK	10/5/15 11:06 AM	shFUBP 1
FUBP1 EM CD41 CD235a 2	OK	10/5/15 11:06 AM	shFUBP 2
FUBP1 EM CD41 CD235a 3	OK	10/5/15 11:07 AM	shFUBP 3
~~TAL1 EM CD41 CD235a 1~~	~~OK~~	~~10/5/15 11:07 AM~~	unrelated experiments
~~TAL1 EM CD41 CD235a 2~~	~~OK~~	~~10/5/15 11:07 AM~~	deleted
~~TAL1 EM CD41 CD235a 3~~	~~OK~~	~~10/5/15 11:07 AM~~	

FACSDiva Version 6.1.3

wild type unstained

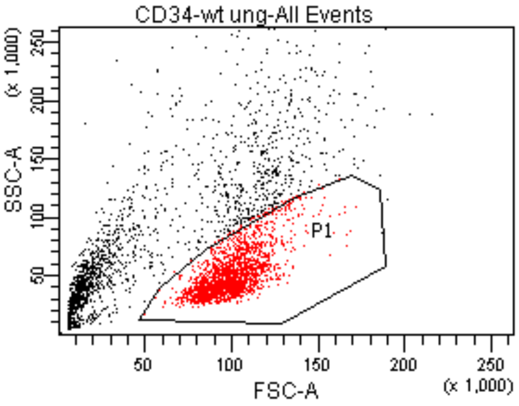

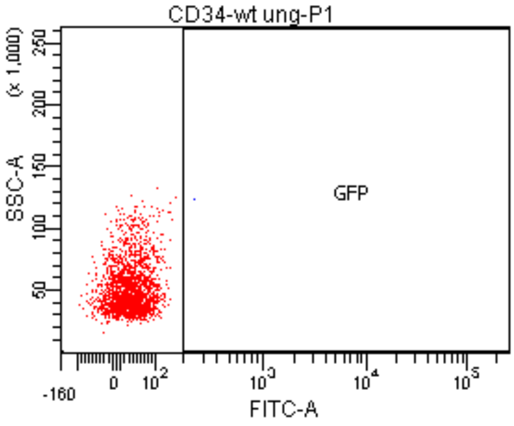

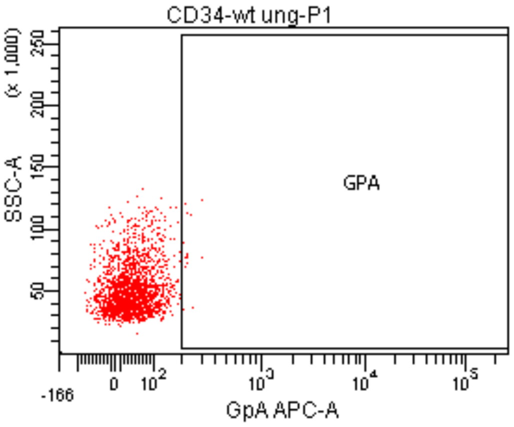

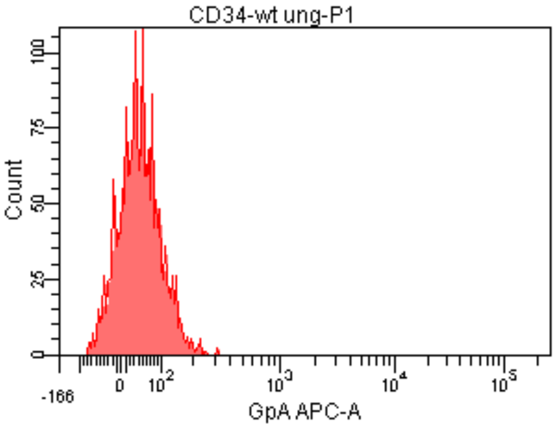

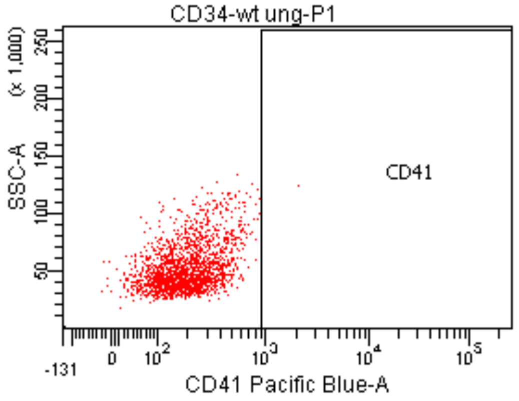

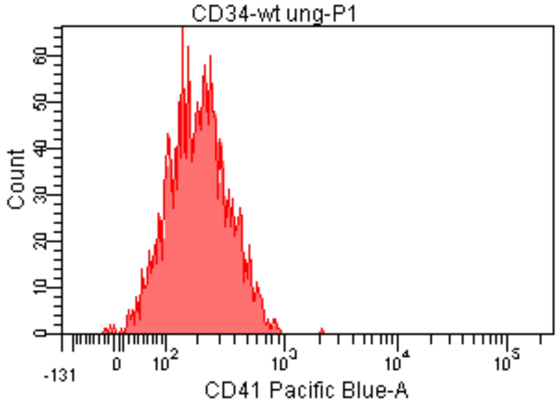

Tube: wt ung

Population
- All Events
  - P1
    - GFP
      - GPA
      - CD41

Experiment Name: 20151005 CD34 FUBP1 TAL1 EM ...
Specimen Name: CD34
Tube Name: wt ung
Record Date: Oct 5, 2015 10:34:56 AM

Population	#Events	%Parent	FITC-A Mean	FITC-A Median	GpA AP... Mean	GpA AP... Median
All Events	####	####	46	39	103	46
P1	####	####	33	32	42	38
GFP	1	0.0	252	252	309	309
GPA	1	100.0	252	252	309	309
CD41	1	100.0	252	252	309	309

FACSDiva Version 6.1.3

transduced unstained

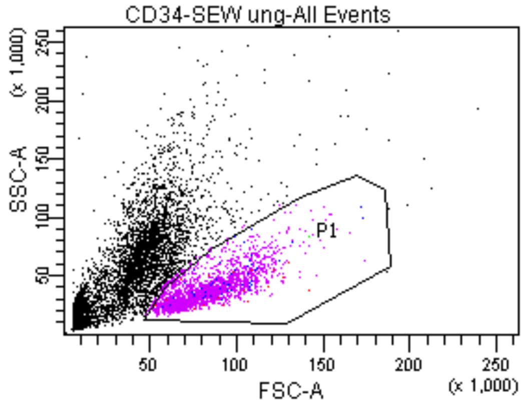

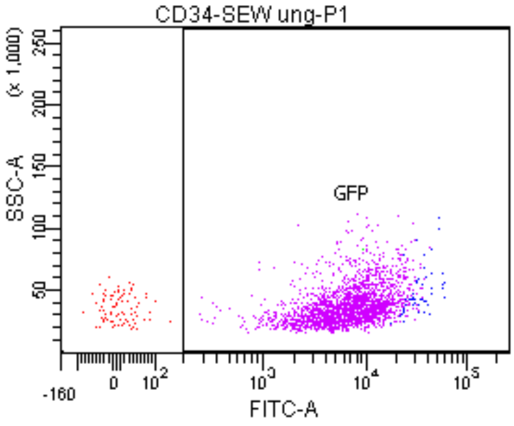

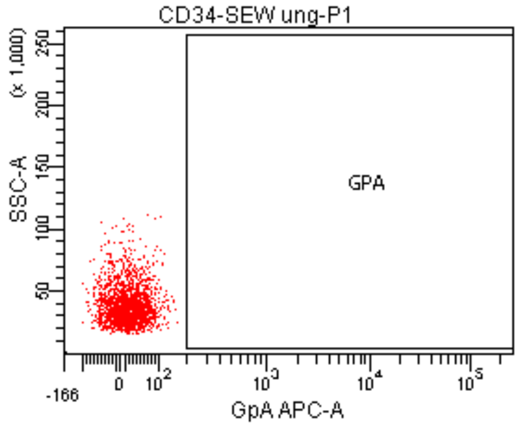

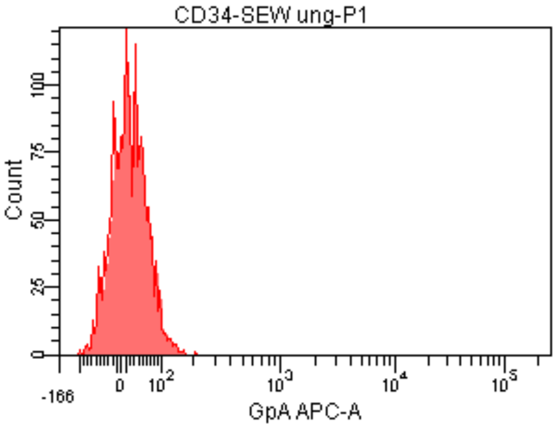

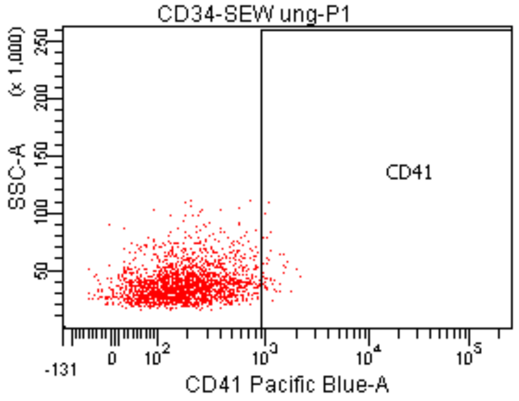

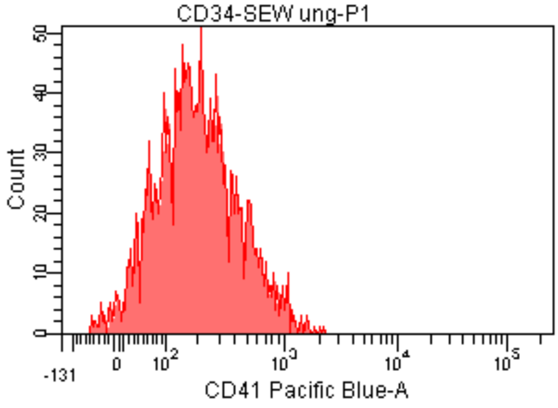

Tube: SEW ung

Population
- All Events
  - P1
    - GFP
      - GPA
      - CD41

Experiment Name: 20151005 CD34 FUBP1 TAL1 EM ...
Specimen Name: CD34
Tube Name: SEW ung
Record Date: Oct 5, 2015 10:36:53 AM

Population	#Events	%Parent	FITC-A Mean	FITC-A Median	GpA AP... Mean	GpA AP... Median
All Events	####	####	5,942	2,879	70	22
P1	####	####	9,081	7,154	17	15
GFP	2,000	95.8	9,476	7,514	17	15
GPA	1	0.0	9,594	9,594	202	202
CD41	48	2.4	36,933	34,190	7	6

unrelated experiment

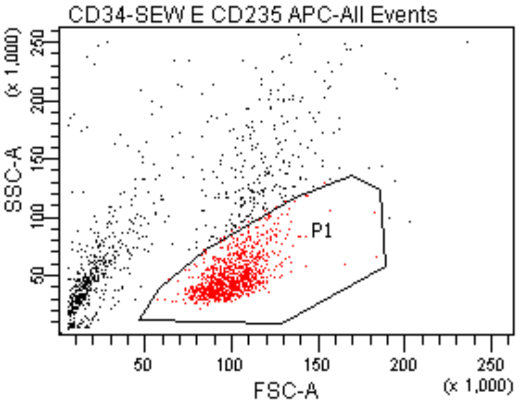

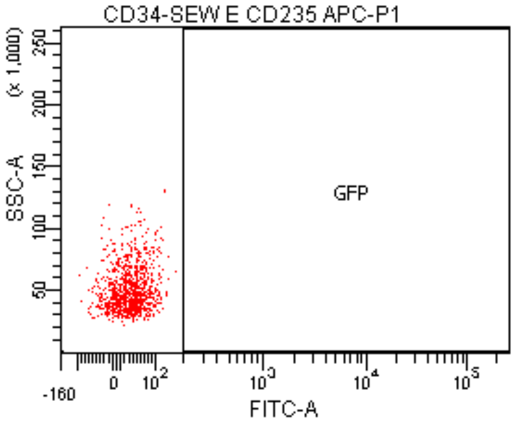

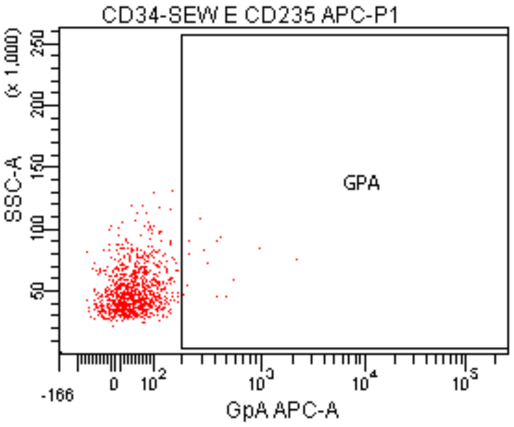

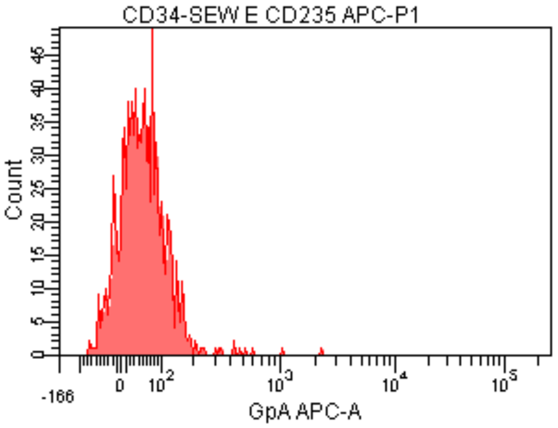

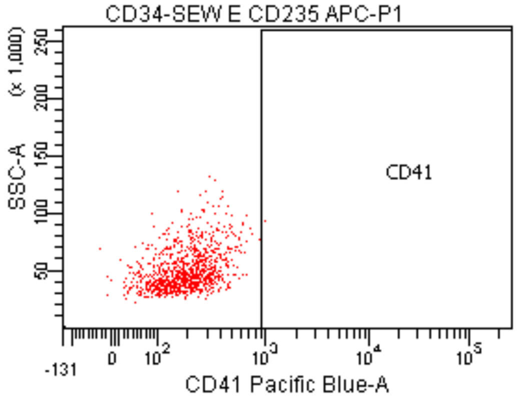

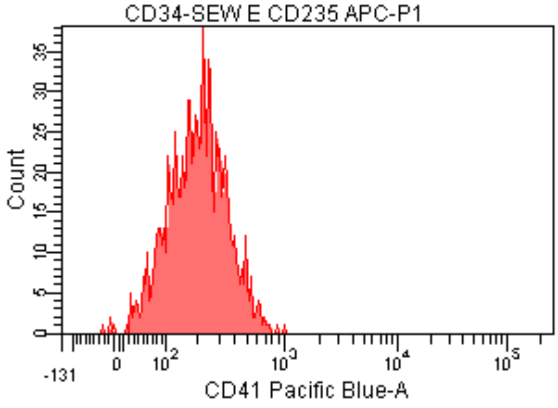

Tube: SEW E CD23...

Population
- All Events
  - P1
    - GFP
      - GPA
      - CD41

Experiment Name: 20151005 CD34 FUBP1 TAL1 EM ...
Specimen Name: CD34
Tube Name: SEW E CD235 APC
Record Date: Oct 5, 2015 10:38:43 AM

Population	#Events	%Parent	FITC-A Mean	FITC-A Median	GpA AP... Mean	GpA AP... Median
All Events	####	####	49	39	125	50
P1	####	####	33	34	53	43
GFP	0	0.0	####	####	####	####
GPA	0	####	####	####	####	####
CD41	0	####	####	####	####	####

unrelated experiment

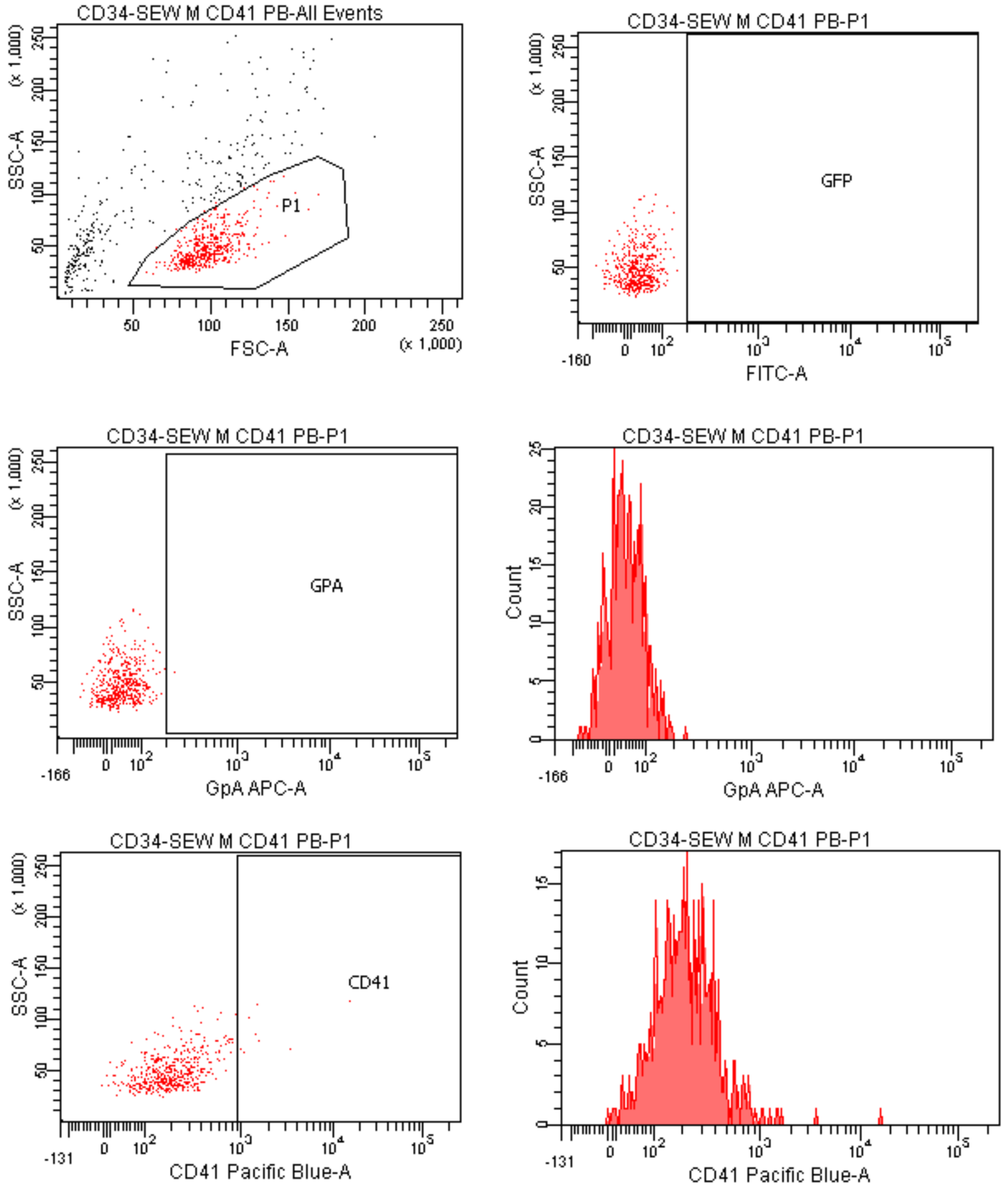

Tube: SEW M CD41...

Population
- All Events
  - P1
    - GFP
      - GPA
      - CD41

Experiment Name:	20151005 CD34 FUBP1 TAL1 EM ...
Specimen Name:	CD34
Tube Name:	SEW M CD41 PB
Record Date:	Oct 5, 2015 10:39:36 AM

Population	#Events	%Parent	FITC-A Mean	FITC-A Median	GpA AP... Mean	GpA AP... Median
All Events	####	####	47	37	125	46
P1	####	####	29	27	40	36
GFP	0	0.0	####	####	####	####
GPA	0	####	####	####	####	####
CD41	0	####	####	####	####	####

FACSDiva Version 6.1.3

shcontrol 1

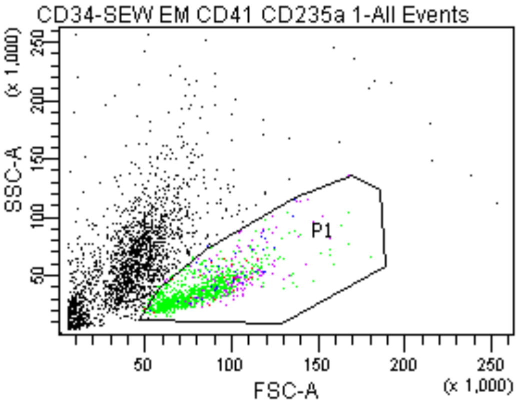

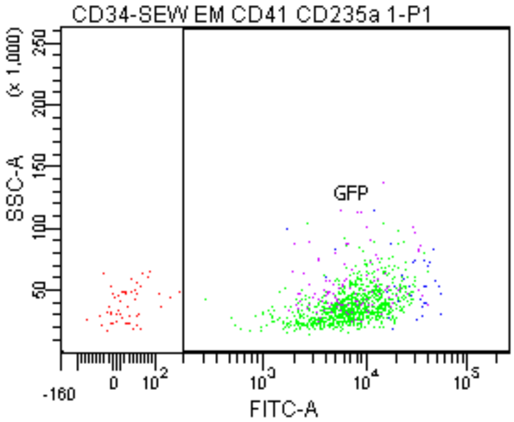

CD235a GYPA

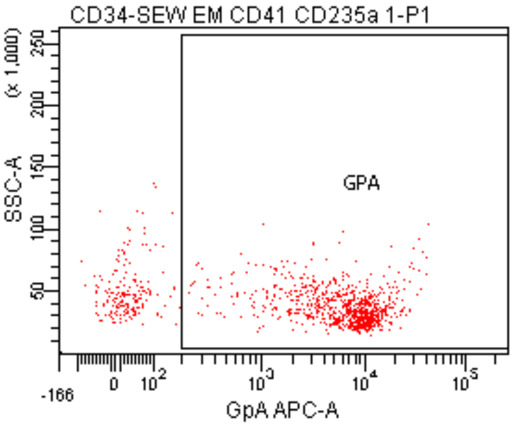

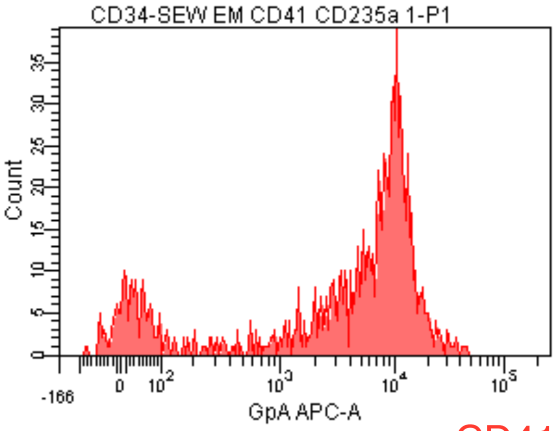

CD41

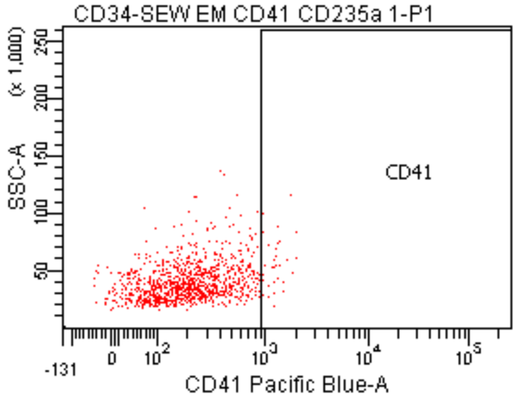

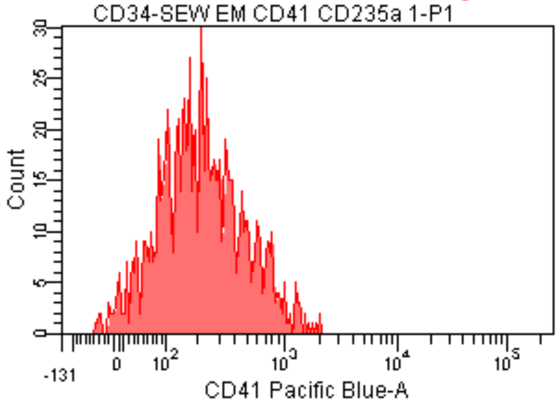

Tube: SEW EM CD4...

Population
- All Events
  - P1
    - GFP
      - GPA
      - CD41

Experiment Name:	20151005 CD34 FUBP1 TAL1 EM ...
Specimen Name:	CD34
Tube Name:	SEW EM CD41 CD235a 1
Record Date:	Oct 5, 2015 10:40:57 AM

Population	#Events	%Parent	FITC-A Mean	FITC-A Median	GpA AP... Mean	GpA AP... Median
All Events	####	####	5,942	2,817	3,595	1,568
P1	####	####	9,328	7,256	7,067	6,719
GFP	1,000	95.1	9,803	7,478	7,210	6,838
GPA	829	82.9	9,677	7,651	8,691	8,357
CD41	32	3.2	31,387	32,280	6,158	4,028

shcontrol 2

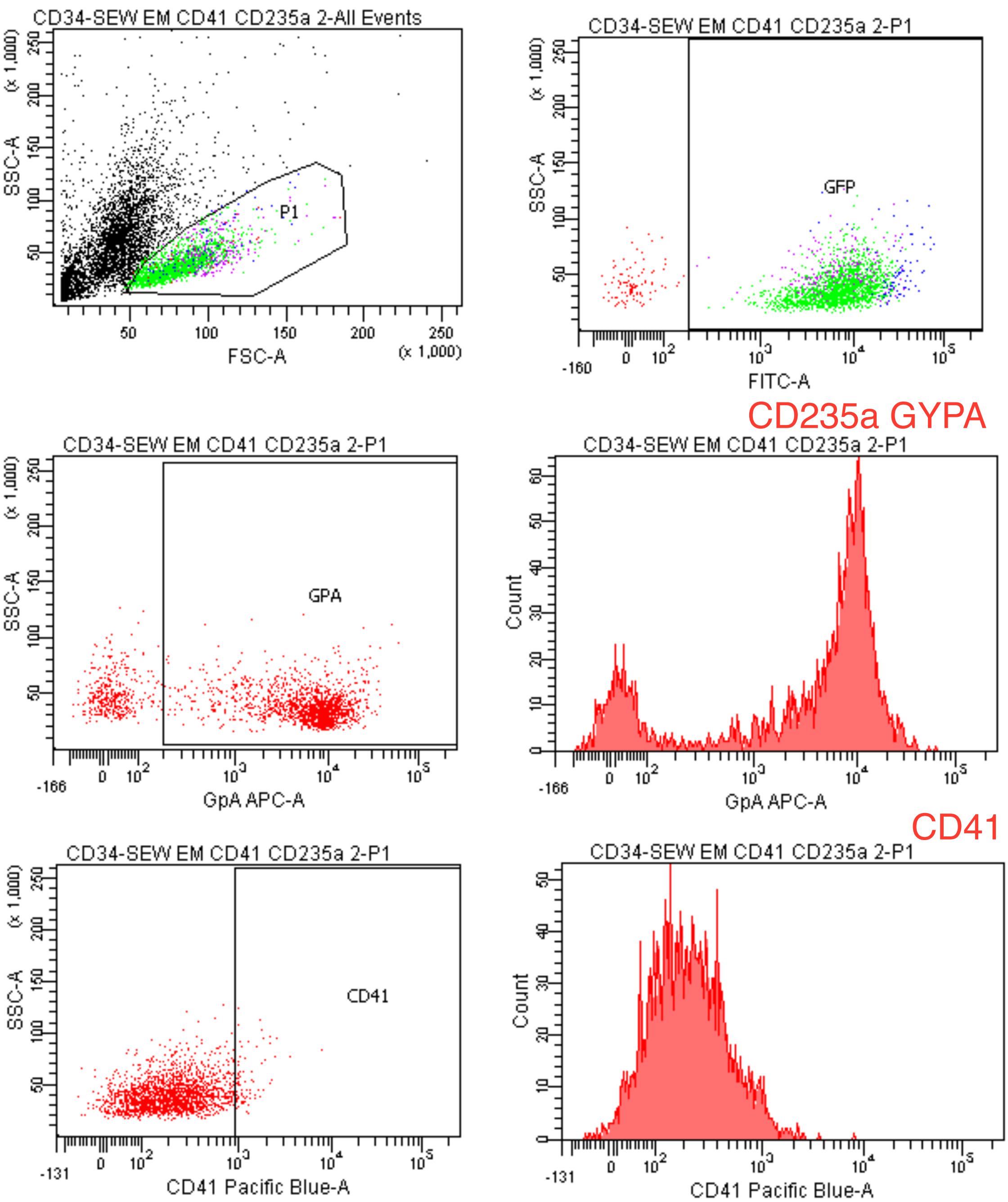

Tube:	SEW EM CD4...
Population	
All Events	
P1	
GFP	
GPA	
CD41	

Experiment Name:	20151005 CD34 FUBP1 TAL1 EM ...
Specimen Name:	CD34
Tube Name:	SEW EM CD41 CD235a 2
Record Date:	Oct 5, 2015 10:43:38 AM

Population	#Events	%Parent	FITC-A Mean	FITC-A Median	GpA AP... Mean	GpA AP... Median
All Events	####	####	5,565	2,233	3,413	1,361
P1	####	####	9,413	7,339	6,886	6,570
GFP	2,000	95.3	9,874	7,699	6,996	6,684
GPA	1,623	81.2	9,675	7,739	8,614	8,242
CD41	80	4.0	34,107	32,347	6,190	3,900

shcontrol 3

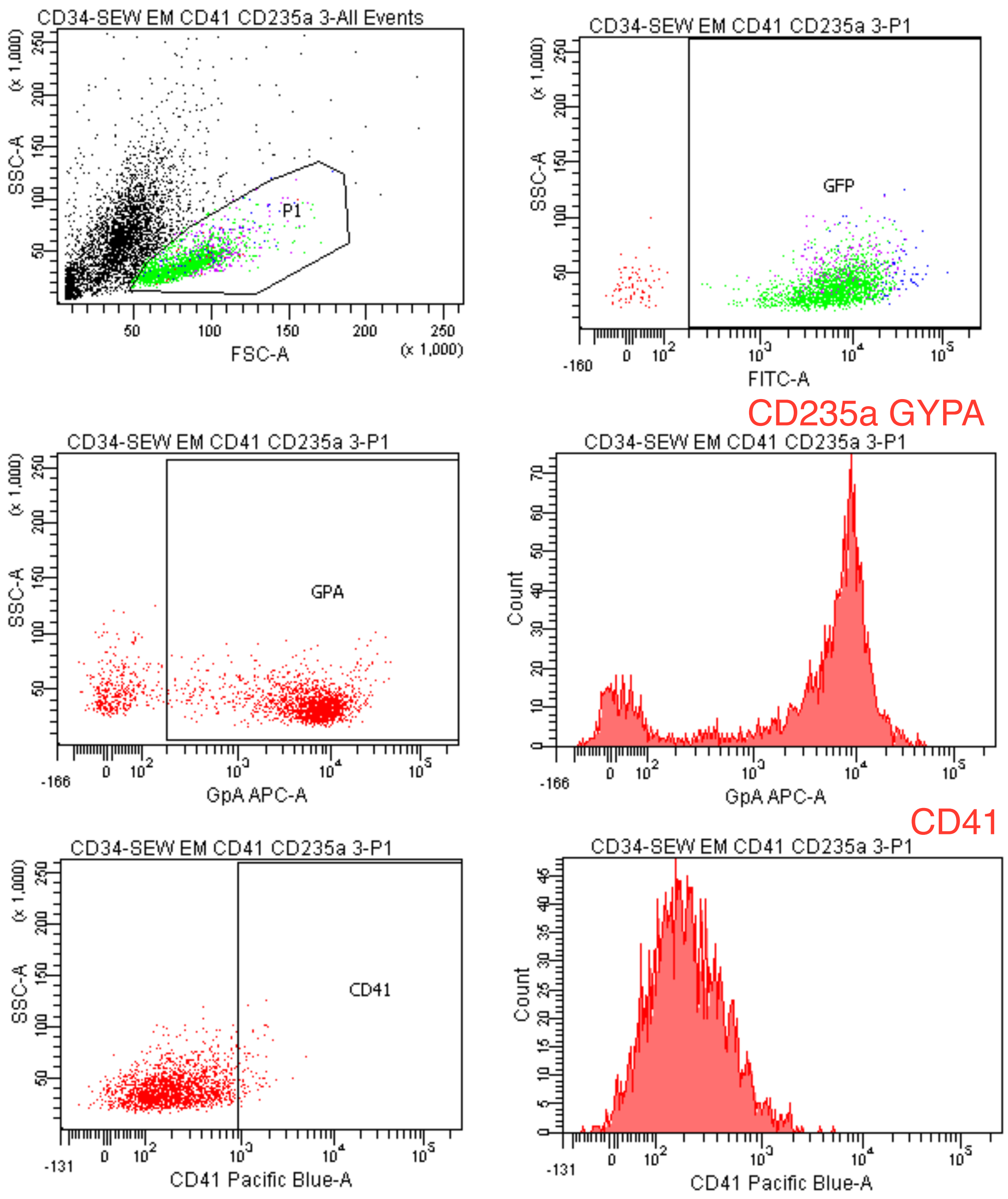

Tube: SEW EM CD4...

Population
- All Events
  - P1
    - GFP
      - GPA
      - CD41

Experiment Name:	20151005 CD34 FUBP1 TAL1 EM ...
Specimen Name:	CD34
Tube Name:	SEW EM CD41 CD235a 3
Record Date:	Oct 5, 2015 10:45:53 AM

Population	#Events	%Parent	FITC-A Mean	FITC-A Median	GpA AP... Mean	GpA AP... Median
All Events	####	####	5,620	2,733	3,314	1,499
P1	####	####	8,998	6,954	6,423	6,194
GFP	2,000	96.4	9,335	7,205	6,499	6,311
GPA	1,653	82.6	8,896	7,057	7,858	7,438
CD41	65	3.2	33,669	30,541	4,814	1,449

FACSDiva Version 6.1.3

shFUBP1 1

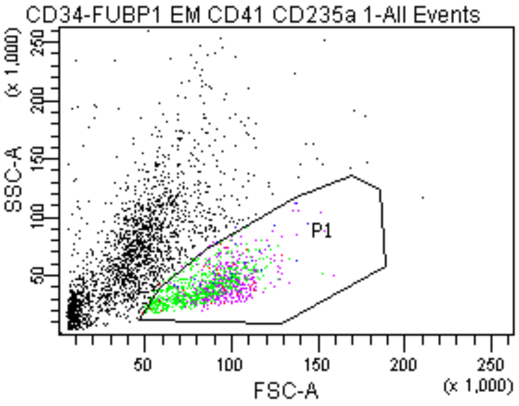

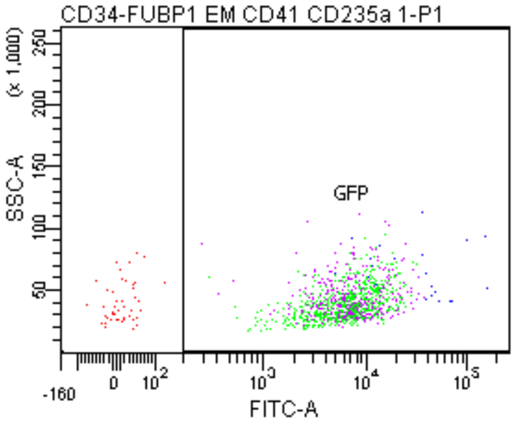

CD235a GYPA

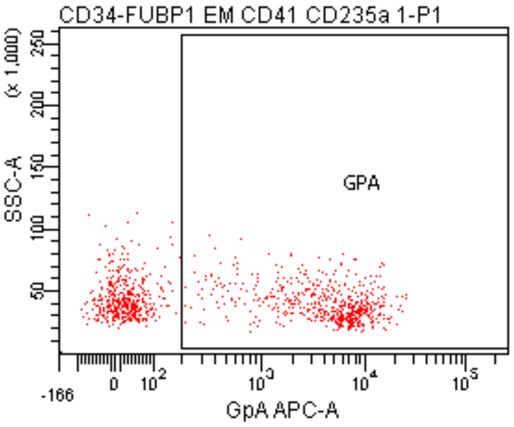

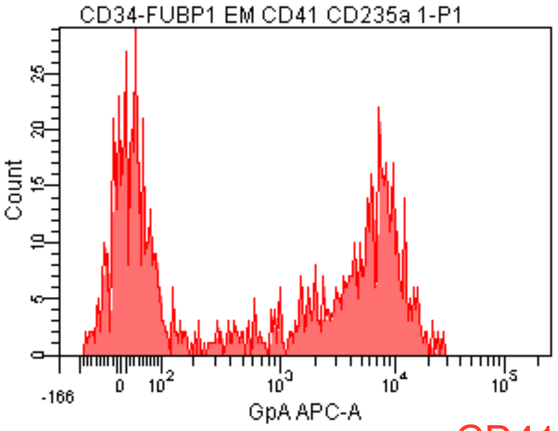

CD41

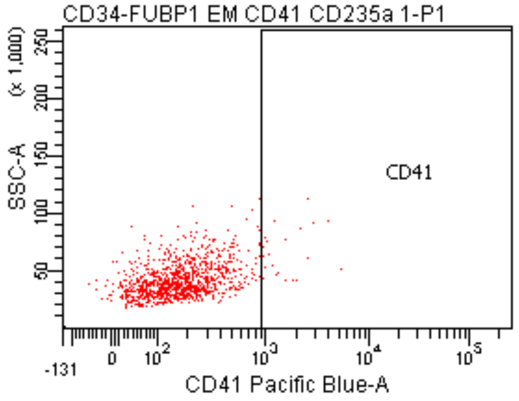

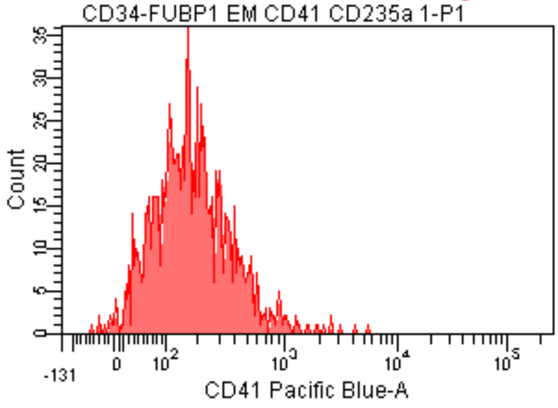

Tube: FUBP1 EM C...

Population
- All Events
  - P1
    - GFP
      - GPA
      - CD41

Experiment Name:	20151005 CD34 FUBP1 TAL1 EM ...
Specimen Name:	CD34
Tube Name:	FUBP1 EM CD41 CD235a 1
Record Date:	Oct 5, 2015 10:48:04 AM

Population	#Events	%Parent	FITC-A Mean	FITC-A Median	GpA AP... Mean	GpA AP... Median
All Events	####	####	5,674	3,015	1,839	337
P1	####	####	8,804	6,959	3,398	791
GFP	1,000	95.6	9,209	7,196	3,369	679
GPA	547	54.7	8,053	6,916	6,139	5,697
CD41	21	2.1	49,188	40,673	336	38

shFUBP1 2

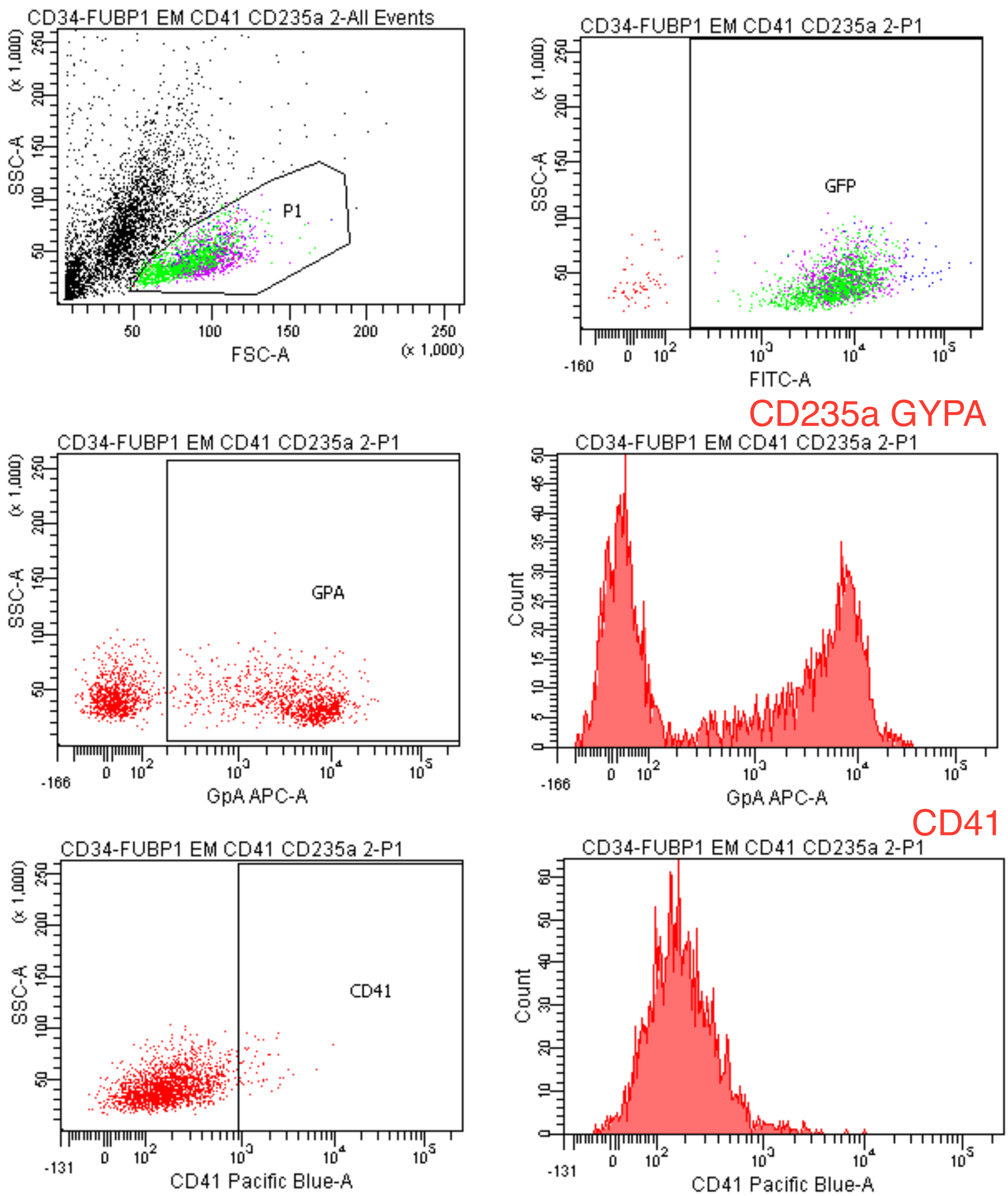

Tube: FUBP1 EM C...

Population
All Events
P1
GFP
GPA
CD41

Experiment Name: 20151005 CD34 FUBP1 TAL1 EM ...
Specimen Name: CD34
Tube Name: FUBP1 EM CD41 CD235a 2
Record Date: Oct 5, 2015 10:54:51 AM

Population	#Events	%Parent	FITC-A Mean	FITC-A Median	GpA AP... Mean	GpA AP... Median
All Events	####	####	5,684	2,797	1,775	269
P1	####	####	9,185	7,254	3,238	658
GFP	2,000	97.1	9,456	7,443	3,221	652
GPA	1,080	54.0	8,111	6,985	5,945	5,443
CD41	45	2.2	49,506	40,169	386	34

shFUBP1 3

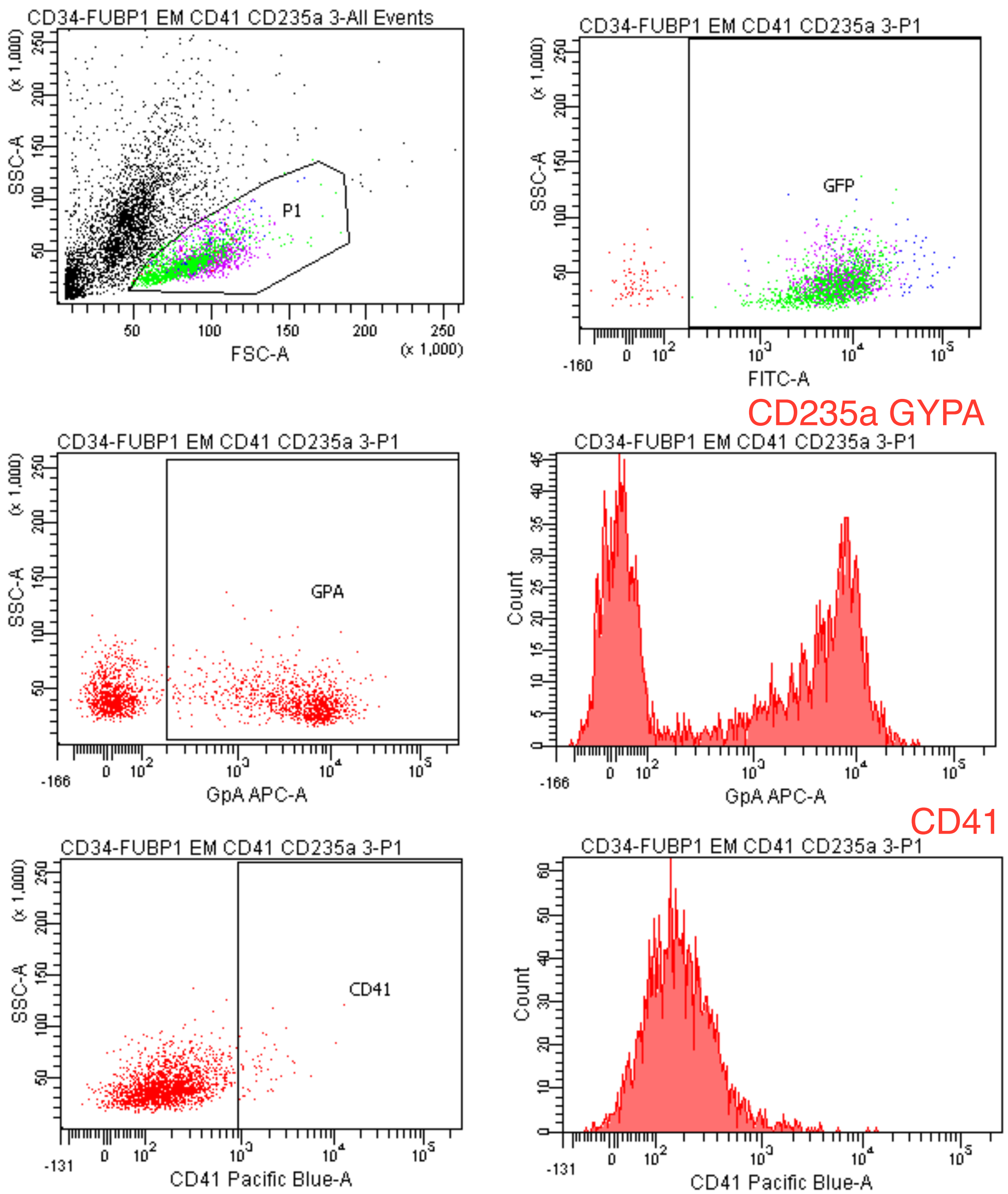

Tube: FUBP1 EM C...

Population

- All Events
  - P1
    - GFP
      - GPA
      - CD41

Experiment Name:	20151005 CD34 FUBP1 TAL1 EM ...
Specimen Name:	CD34
Tube Name:	FUBP1 EM CD41 CD235a 3
Record Date:	Oct 5, 2015 10:51:04 AM

Population	#Events	%Parent	FITC-A Mean	FITC-A Median	GpA AP... Mean	GpA AP... Median
All Events	####	####	5,791	3,167	1,868	309
P1	####	####	8,940	6,933	3,449	991
GFP	2,000	96.5	9,261	7,134	3,431	930
GPA	1,103	55.2	8,068	6,713	6,206	5,646
CD41	51	2.6	44,647	41,238	431	38
