# Supplementary material for: FUSE binding protein 1 (FUBP1) expression is upregulated by T-cell acute lymphocytic leukemia protein 1 (TAL1) and required for efficient erythroid differentiation
Source: PLoS One. 2019 Jan 17;14(1):e0210515. doi: 10.1371/journal.pone.0210515 (PMC6336336; doi:10.1371/journal.pone.0210515)
Supplement: S1 Table — (DOCX) [file pone.0210515.s014.docx]

| oligonucleotide name | method | sequence 5´ → 3´ |
| --- | --- | --- |
| *CD41*  for | RT-qPCR | AATGGCCCCTGCTGTCGTGC |
| *CD41*  rev | RT-qPCR | TGCACGGCCAGCTCTGCTTC |
| *CD71*  for | RT-qPCR | AGGACGCGCTAGTGTTCTTC |
| *CD71*  rev | RT-qPCR | CCAGGCTGAACCGGGTATATG |
| *CD235a*  for | RT-qPCR | CCCTCCAGAAGAGGAAACCGGAGA |
| *CD235a*  rev | RT-qPCR | GGCACGTCTGTGTCAGGTGAGG |
| *FUBP1*  for | RT-qPCR | ACTCCAATGGGACCATACAACCCT |
| *FUBP1*  rev | RT-qPCR | CAGCCCAAGCTGCTGAATTTGGAT |
| *GAPDH*  for | RT-qPCR | TCTTTTGCGTCGCCAGCCGAGC |
| *GAPDH*  rev | RT-qPCR | TGACCAGGCGCCCAATACGACC |
| *GATA1* for | RT-qPCR | GACACTCCCCAGTCTTTCAGG |
| *GATA1* rev | RT-qPCR | CAGTTGAGGCAGGGTAGAGC |
| *GATA2* for | RT-qPCR | GCCGCTCTGTCTTCAAAGTC |
| *GATA2* rev | RT-qPCR | CACAAGATGAATGGGCAGAA |
| *GATA3* for | RT-qPCR | GAACTGTCAGACCACCACAA |
| *GATA3* rev | RT-qPCR | GCCTTCCTTCTTCATAGTCAGG |
| *GATA4* for | RT-qPCR | GGAAGCCCAAGAACCTGAATAA |
| *GATA4* rev | RT-qPCR | TTGCTGGAGTTGCTGGAAG |
| *GATA5* for | RT-qPCR | CACAAGATGAATGGCGTCAAC |
| *GATA5* rev | RT-qPCR | TTGGTCGTGTGGCAGTT |
| *GATA6* for | RT-qPCR | CTCCAACTTCCACCT |
| *GATA6* rev | RT-qPCR | TCGGGATTGGTGCTCTCT |
| *TAL1*  for | RT-qPCR | TCGGCAGCGGGTTCTTTGGG |
| *TAL1*  rev | RT-qPCR | CCATCGCTCCCGGCTGTTGG |
| *FUBP1 P1* for | ChIP-qPCR | TCCTCACGATTCCTGGAGCTTAC |
| *FUBP1 P1* rev | ChIP-qPCR | ACTTCAGTTACCAAGTAATGCCAGA |
| *FUBP1 P2* for | ChIP-qPCR | CACCACCCAAACACAGTCGC |
| *FUBP1 P2* rev | ChIP-qPCR | TTTTGGCACCTCCTCTCCGC |
| *Chromosome 18* for | ChIP-qPCR | ACTCCCCTTTCATGCTTCTG |
| *Chromosome 18* rev | ChIP-qPCR | AGGTCCCAGGACATATCCATT |
| *hGAPDH E6 CHIP for* | ChIP-qPCR | GCCAAGGCTGTGGGCAAGGT |
| *hGAPDH E6 CHIP rev* | ChIP-qPCR | CCTCCGACGCCTGCTTCACC |
