# Supplementary material for: FUSE binding protein 1 (FUBP1) expression is upregulated by T-cell acute lymphocytic leukemia protein 1 (TAL1) and required for efficient erythroid differentiation
Source: PLoS One. 2019 Jan 17;14(1):e0210515. doi: 10.1371/journal.pone.0210515 (PMC6336336; doi:10.1371/journal.pone.0210515)
Supplement: S2 Table — (DOCX) [file pone.0210515.s015.docx]

| antibody | method | dilution | manufacturer |
| --- | --- | --- | --- |
| Anti-CD235a APC | Flow cytometry | 0.015 µg/test | *HIP8, eBioscience* |
| Anti-CD41 APC | Flow cytometry | 0.125 µg/test | *HIR2/GA-R2, eBioscience* |
| Anti-β-Actin | Western Blotting | 1:2000 | *C11, Santa Cruz Biotechnology* |
| Anti-FUBP1 | Western Blotting | 1:2000 | *ab181111, Abcam* |
| Anti-GAPDH | Western Blotting | 1:1000 | *SC-32233*  *Santa-Cruz* |
| Anti-TAL1 | Western Blotting | 1:500 | *04-123, Merck Millipore* |
| Anti-GATA1 | ChIP | 5 µg/IP | *ab181544, Abcam* |
| Anti-RNA Pol II | ChIP | 2.4 µg/IP | *ab26721, Abcam* |
| Anti-TAL1 | ChIP | 5 µg/IP | *TA-590662, Origene* |
